# Sex differences in the association between marital status and the risk of cardiovascular, cancer, and all-cause mortality: a systematic review and meta-analysis of 7,881,040 individuals

**DOI:** 10.1186/s41256-020-00133-8

**Published:** 2020-02-28

**Authors:** Yafeng Wang, Yurui Jiao, Jing Nie, Adrienne O’Neil, Wentao Huang, Lei Zhang, Jiafei Han, Hao Liu, Yikun Zhu, Chuanhua Yu, Mark Woodward

**Affiliations:** 1 Department of Epidemiology and Biostatistics, School of Health Sciences, Wuhan University, 185 Donghu Road, Wuchang District, Wuhan, 430071 China; 2 Department of endocrinology, The Second Hospital of Shanxi Medical University, Taiyuan, China; 3 Department of Sociology & Institute for Empirical Social Science Research, School of Humanities and Social Sciences, Xi’an Jiaotong University, Xi’an, China; 4 Melbourne School of Population and Global Health, University of Melbourne, Carlton, Australia; 5 School of Nursing, Guangdong Pharmaceutical University, Guangzhou, China; 6 Shanghai Institute of Cardiovascular Diseases, Zhongshan Hospital of Fudan University, Shanghai, China; 7 Faculty of Psychology, Beijing Normal University, Beijing, China; 8 Department of Ophthalmology, The First People’s Hospital of Xianyang City, Xianyang, China; 9 The George Institute for Global Health, University of Oxford, Oxford, UK; 10 The George Institute for Global Health, University of New South Wales, Sydney, Australia; 11 Department of Epidemiology, Johns Hopkins University, Baltimore, MD USA

**Keywords:** Marital status, Sex difference, Mortality, Meta-analysis

## Abstract

**Purpose:**

To ascertain whether sex differences exist in the relationship between marital status and cardiovascular diseases (CVD), coronary heart disease (CHD), cancer and all-cause mortality in the general population and to explore the potential effect of age, location, the duration of follow-up and publication years on these outcomes.

**Methods:**

A systematic search was performed in PubMed and EMBASE from inception through to April 2018 and review of references to obtain sex-specific relative risks and their 95% confidence intervals. These were used to derive the women-to-men ratio of RRs (RRR) and 95% CI for each study. RRs and RRRs for each outcome were then pooled using random effects inverse-variance weighted meta-analysis.

**Results:**

Twenty-one studies with 7,891,623 individuals and 1,888,752 deaths were included in the meta-analysis. Compared with married individuals, being unmarried was significantly associated with all-cause, cancer, CVD and coronary heart disease mortalities for both sexes. However, the association with CVD and all-cause mortality was stronger in men. Being divorced/separated was associated with a higher risk of all-cause mortality in men and a stronger risk of cancer and CVD mortality. The pooled ratio for women versus men showed 31 and 9% greater risk of stroke mortality and all-cause mortality associated with never married in men than in women.

**Conclusions:**

Being unmarried conferred higher risk of stroke and all-cause mortality for men than women. Moreover, divorced/separated men had higher risk of cancer mortality and CVD mortality. Further studies are warranted to clarify the biological, behavioral, and/or social mechanisms involved in sex differences by these associations.

## Background

Marital status has been identified as an important social factor associated with mortality. In current epidemiologic research, being unmarried was observed to be a suboptimal health status in the global population [1]. In 2017, more than 45% of Americans were unmarried and approximately 35.25 million people were living alone [2]. In China, the population of unmarried people has also steadily increased; reaching up to 218 million in the end of 2016, of which 129 million were men and 89 million were women [3]. The growing number of unmarried people has health implications, in light of evidence suggesting that it is associated with an increase in the incidence of various diseases and high mortality.

The beneficial effect of marriage on health is one of the most consistently positive findings in medical sociology and epidemiology [4]. Marriage offers a direct form of social support [5, 6] and it can reduce the risk of unhealthy behaviors such as poor diet or alcohol use [5–7]. In contrast, being unmarried has been suggested to contribute to less intimate social networks, loneliness and increased levels of stress hormones [8], which may increase risk from cardiovascular diseases (CVD) [9, 10], cancer [11, 12] or metabolic syndrome [13, 14]. While the links between marital status and CVD mortality risk have been widely reported [15–19], comparatively less has been observed regarding other conditions including cancer mortality and all-causes more broadly.

What also remains unclear is whether (and to what degree) specific types of non-marital statuses (widowed, divorced/separated or never married) are differentially associated with the aforementioned outcomes. Explicating these links are likely to be important in understanding the mechanisms that might underpin the relationship between marital status and disease risk, particularly as societal trends and attitudes towards marital status change. What is more, few attempts have been made to produce an overall estimate and sufficiently clarified of the sex difference between unmarried status and mortality risks, this is still a matter of debate. Evidence shows that men tend to benefit more from being married than do women with respect to their health [20–24]. A prospective register study of Finnish men and women found that living alone was a predictor of CVD mortality for men, while risk was higher for women who cohabitated [25]. Thus, it stands to reason that men would experience the greatest health losses in the absence of, or dissolution, of a marriage.

Therefore, we performed a meta-analysis of prospective cohort studies to ascertain the sex difference between marital status and CVD, cancer, all-cause mortality in the general population and to explore the potential effect of age, location, the duration of follow-up and publication years on these outcomes.

## Methods

This meta-analysis was conducted in accordance with the MOOSE (Meta-analysis Of Observational Studies in Epidemiology) guidelines [26].

### Literature search strategy

A systematic search was performed in PubMed and EMBASE from their inception (1966 and 1947, respectively) through to April 2018 using the key words “marital status”, “married”, “unmarried”, “widowed”, “divorced”, “single”, “separated”, “mortality” and “prospective cohort studies”. The detailed search strategies were shown in the Supplemental Material. Only papers published in English language were considered. In addition, we also scrutinized the references of all identified reports for other potentially relevant publications and relevant reviews. If the information of the studies were incomplete, we also contacted the author to obtain sufficient data.

### Study selection

Studies were included in this meta-analysis that met the following inclusion criteria: (1) Being a prospective cohort study; (2) Evaluating the association between marital status and mortality by sex; (3) The included studies had at least two groups pertaining to marital status (e.g married and not married), and the marital status of those non-married was defined by a “no” response to the question, “Have you ever been married?”, which included divorced/separated, widowed and never married (4) The outcomes of the studies included at least one of the following: all-cause mortality, cancer mortality, CVD mortality, CHD mortality and/or stroke mortality; (5) Providing information about the multiple-adjusted risk ratio (RR), odds ratio (OR) and hazard ratio (HR) as well as corresponding 95% confidence interval (CI) of the association between marital status and mortality in men and women; (6) If more than one article was published that based on the same population, we included only the article that provided results with most recent data and the largest number of participants. The exclusion criteria were as follows: (1) studies that were matched cohort design; (2) the results of the studies were not adjusted for at least age. Moreover, we also used individual participant data from the US National Health Interview Surveys (1997 to 2009) which linked National Death Index records through December 31, 2011.

### Data extraction and risk of bias assessment

Details on study characteristics (first author’s last name, publication year, location and ethnicity, study design, duration of follow-up), information regarding the included population (population source, number of participants with the martial status of married and not married, mean age, number of men and women) and the data on the outcomes [(all-cause mortality, cancer mortality, CVD mortality, CHD mortality, and stroke mortality), ICD code (if available)] were extracted by two authors (YR J and JF H) from each identified study by using a standardized extraction sheet independently, with disagreements resolved by discussion. We also extracted sex-specific multiple-adjusted measures of relative risk (RR; or equivalents) and 95% confidence intervals.

The quality of each study was estimated according to the NOS (Newcastle-Ottawa Quality Assessment Scale) [27], which consists of 3 variables of quality as follows: object selection (4 points), comparability (2 points), and exposure and outcome (3 points) and each satisfactory answer received one star. Nine stars represents the best. We considered the studies with a score of ≥6 reflecting high quality, while ≤4 to be of low quality (Supplement Table 1).

### Statistical analysis

For each study, we obtained the sex-specific RR or equivalents for individuals who were not married or subcategories of the unmarried (i.e. divorced/separated, widowed or never married) vs individuals who were married and 95% CIs. We log transformed these RRs or equivalents and computed women-to-men ratio of RRs (RRR) and 95% CIs to compare the sexes directly. These RRRs were calculated for studies with multiple-adjusted estimates [28]. We subsequently pooled the differences across studies using random-effects meta-analysis weighted by the inverse of the variances of the log RRRs, and then back transformed the data to obtain the pooled women-to-men ratio of the RR (RRR). In addition, for the individual participant data from the NHIS (1997 to 2009) linked mortality data, we also assessed the RRs, women-to-men ratio of RRs (RRR) and 95% CIs used the same method. We also pooled relative risks for men and women separately. For one study, which reported separate hazard ratios for men and women in different divorced/separated, widowed and single groups, we first used inverse variance weighted random-effects meta-analysis to generate a summary hazard ratio of not married or the subsets of unmarried for men and for women. In addition, only one or two of three types of marital status category (i.e. divorced/separated, widowed or never married), could not be combined as the estimates of not married. Heterogeneity between studies was evaluated by using the Q test and I^2^ statistic. The level of significance for the Q test was defined as *P* < 0.10. I^2^ statistic was used to estimate the percentage of variability between studies due to between-study heterogeneity. I^2^ values ≤50 and > 50% indicated no and significant heterogeneity respectively [29, 30].

Sensitivity analyses were performed by location (Asian, European, American and others), mean age groups (< 60 vs ≥60 years), follow-up duration (< 10 vs ≥10 years) and publication years (pre-2000, 2001–2010, post-2010) and by sex. Random-effects meta-regression analyses were used to assess whether the differences in the mean baseline age and mean follow-up duration contributed to heterogeneity among the studies. We used the Egger’s test, Begg’s test and funnel plots (of the natural log of the RRR against its standard error) to examine publication bias for all primary analyses, and trim and fill analysis to adjust the RRRs for the presence of publication bias when more than 5 articles were included [31]. All analyses were performed using Stata version 12.0. Two-sided *P* value smaller than 0.05 was considered statistically significant.

## Results

### Study characteristics

**Fig. 1:**
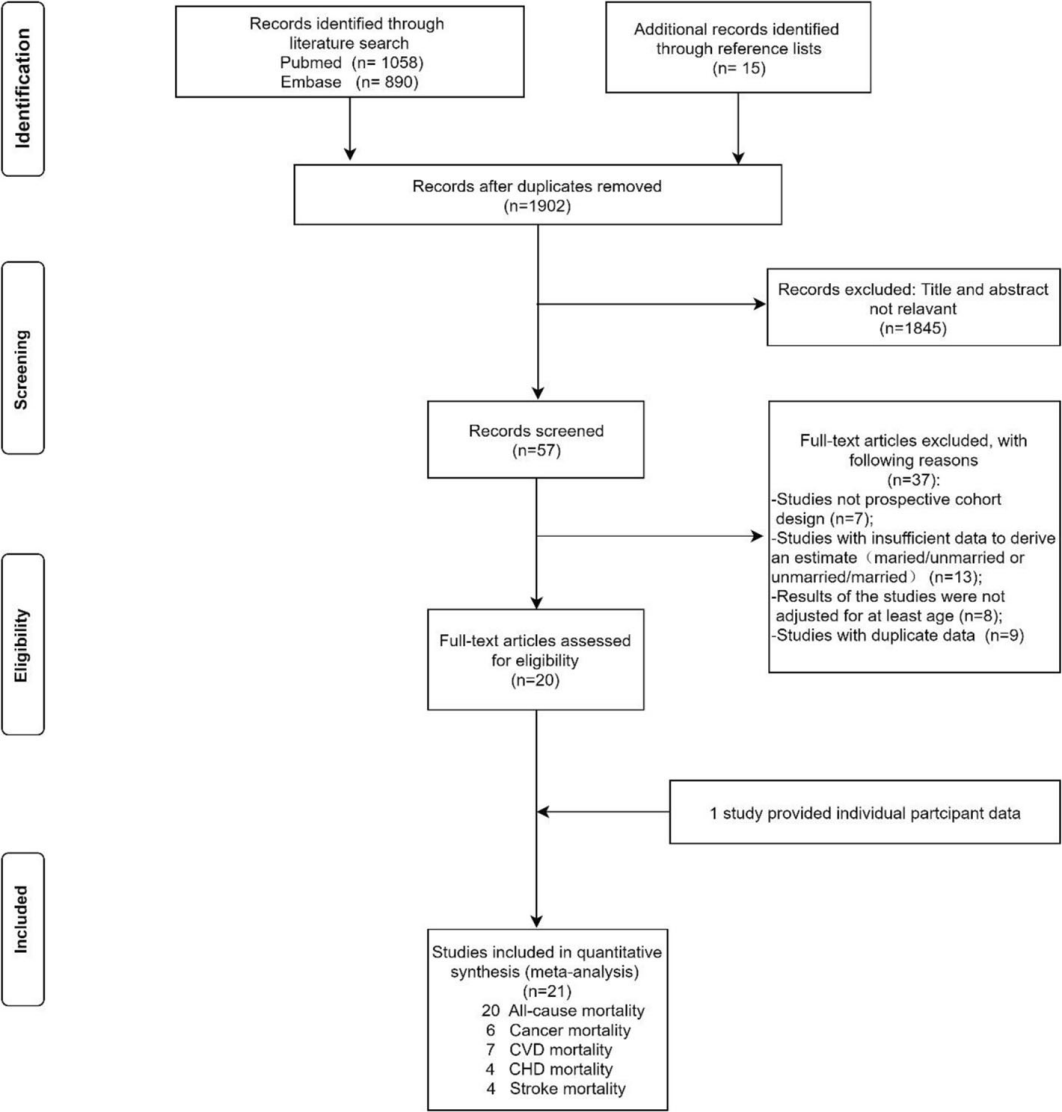
Flowchart for study selection for the meta-analysis

The flowchart of the process of study selection is shown in Fig. 1. Overall, 21 studies [20–22, 32–48] (20 studies were retained for inclusion and one additional study was provided with individual participant data) comprising 7,881,040 individuals were analyzed. The number of participants ranged from 3, 386 to 6,500,000 among the studies, which were performed in 15 countries (9 studies from Europe, 6 from Asia, and 6 from America). Moreover, 20 studies reported data on all-cause mortality (7,846,939 participants, 1,887,151 deaths), 6 studies on cancer mortality (7,081,927 participants, 165,826 deaths), 7 studies on CVD mortality (7,095,655 participants, 128,961 deaths), 5 studies on CHD mortality (288,719 participants, 15,140 deaths), and 5 studies on stroke mortality (583, 148 participants, 49,393 deaths). Individuals included in these studies were aged between 42.1 and 72.9 years old at baseline and the duration of study follow-up ranged from 5 to 29 years. The main characteristics of the included studies are summarized in Table 1.

**Table 1:** Characteristics of studies included in the meta-analysis

Author	Study location	Study name	No. of participants	No. of death	Mean Age	Reference	Unmarried status category	Outcomes	Mean/medium follow up (years)	Maximum adjustment available
Va et al, 2011 [32]	China	Shanghai Women’s Health Study; Shanghai Men’s Health Study	127,004	4,116	53.8	Married	Not married Never married Widowed Divorced/separated	All-cause mortality Cancer mortality CVD mortality CHD mortality Stroke mortality	NA	Age, income, education, occupation, BMI, WHR, physical activity, smoking, alcohol drinking, ginseng use, HRT, menopause, women only, total energy intake, vegetable and fruit intake, and red meat intake, hypertension, diabetes, baseline CVD, and all other chronic disease
Frisch et al, 2013 [33]	Denmark	Civil Registration System	6,500,000	1,709,850	NA	Married	Never married Widowed Divorced/separated	All-cause mortality Cancer mortality CVD mortality	29	Age, municipality, population density, educational level, and relative income two years before the actual year
Molloy et al, 2009 [35]	Scotland	Scottish Health Survey	13,880	892	52.3	Married	Never married Widowed Divorced/separated	All-cause mortality CVD mortality	7.1	Age
Dupre et al, 2009 [36]	USA	Health and Retirement Study	8,715	1,531	55.9	Married	Widowed Divorced/separated	All-cause mortality	9.4	Age, ethnicity, region, household size, children, education, occupational status, occupational tenure, wealth, health insurance coverage, smoking, alcohol consumption, physical exercise, BMI, preventive health care, hypertension, diabetes, cancer, lung disease, heart disease, stroke, any ADL limitations, depressive symptoms
Scafato et al, 2006 [37]	Italian	Italian Longitudinal Study on Aging	4,514	1,977	72.6	Married	Not married	All-cause mortality	5.8	Age, SBP, DBP, glucose, cholesterol, HDL-CH, BMI, education, procreation, smoking habit, alcohol use, ADLs, IADLs, depression and cognitive impairment
Stimpson et al, 2008 [38]	Mexico	Epidemiologic Studies of the Elderly, 1993–2000 (Hispanic EPESE)	3,386	441	65	Married	Widowed	All-cause mortality	7	Age, education, US nativity, financial strain, social support, health behaviors, medical conditions, disability, alcohol consumption, smoking, BMI, chronic conditions, lower body mobility, and depressive symptoms
Ikeda et al, 2007 [39]	Japan	Japan Collaborative Cohort Study	94,062	8,365	56.8	Married	Never married Widowed Divorced/separated	All-cause mortality Cancer mortality CVD mortality CHD mortality Stroke mortality	9.9	Age, BMI, smoking status, alcohol intake, education, minutes of walking, hours of doing sports, employment status, stress, having children, history of hypertension, and history of diabetes
Eaker et al, 2007 [20]	USA	The Framingham Offspring Study	3,682	NA	48.5	Married	Not married	All-cause mortality	3	Age, SBP, BMI, smoking, diabetes, total/high-density cholesterol
Jaffe et al, 2005 [41]	Israel	Israel Longitudinal Mortality Study	131,156	2,7334	62.2	Married	Not married	All-cause mortality	9.5	Age, Individual income, race, smoking, alcohol consumption, body mass index, and perceived health status
Nilsson et al, 2005 [42]	Sweden	The Malmo Preventive Project	33,346	7,115	45.5	Married/cohabiting	Not married Widowed Divorced/separated	All-cause mortality	9.5	Age and social class
Hurt et al, 2003 [43]	Bangladesh	Centre for Health and Population Research (ICDDR)	29,606	1,233	46.5	Married	Widowed Divorced/separated	All-cause mortality	16	Age, time, parity, own education, wife's education, religion, male occupation; area of residence, percentage of children surviving
Strand et al, 2004 [44]	Norway	NA	44,684	6,506	42.1	Married	Not married Widowed Divorced/separated	CHD mortality	26	Age, smoking, physical activity, BMI, diastolic blood pressure, systolic blood pressure, cholesterol
Malyutina et al, 2004 [45]	the Soviet Union	Novosibirsk MONICA Project	11,404	1,602	NA	Married	Not married Never married Widowed Divorced/separated	All-cause mortality CVD mortality CHD mortality Stroke mortality	15.2	Age, smoking, total cholesterol, systolic blood pressure, frequency of drinking, and BMI; total cholesterol, systolic blood pressure, frequency of drinking, BMI, education
Nagata et al, 2003 [46]	Japan	NA	3,505	678	70.9	Married	Never married Widowed	All-cause mortality	7	Age, number of children, years of education, smoking status, occupation (administrative/professional and the others), and alcohol intake
Johnson et al, 2000 [47]	USA	The National Longitudinal Mortality Study	281,460	1,5364	58.3	Married	Never married Widowed Divorced/separated	All-cause mortality	11	Age, Income, education, labor force status
Iwasaki et al, 2002 [48]	Japan	The Komo-Ise Study	11,565	490	NA	Married	Never married Widowed Divorced/separated	All-cause mortality Cancer mortality CHD mortality Stroke mortality	7	Age, area, occupation, educational background, smoking habit, alcohol consumption, BMI, chronic disease for men and these variables except for alcohol consumption for women
Breeze et al, 1999 [21]	UK	The Longitudinal Study	93,931	63,405	63.7	Married/cohabiting	Never married Widowed Divorced/separated	All-cause mortality	10	Age, social class, tenure and car availability
Fuhrer et al, 1999 [22]	France	Personness Agées Quid (PAQUID) study	3,777	849	73.2	Married	Not married	All-cause mortality	5	Age, education, IADL, cognitive function, hospitalization in preceding year, smoking habits, alcohol consumption
Smith et al, 1997 [40]	USA	NA	10,183	2,633	50.2	Married	Not married	All-cause mortality Cancer mortality CVD mortality	14	Age, race, alcohol consumption, serum cholesterol, hypertension status, BMI, baseline health, education, smoking status, physical exercise, household-level poverty
Nilsson et al, 1998 [34]	Sweden	The Swedish Annual Level-of-Living Survey (SALLS)	39,055	4,897	48.1	Married/cohabiting	Never married	All-cause mortality	16	Age, educational level, marital status, form of tenure, car ownership
NHIS, 2018	USA	National Health Interview Survey	339,113	62,245	45.3	Married	Never married Widowed Divorced/separated	All-cause mortality Cancer mortality CVD mortality Stroke mortality	8.4	Age, sex, race, education, marital status, BMI, smoking, physical activity, drink, hypertension, diabetes, heart disease, stroke, cancer

Abbreviations: *BMI* body mass index; *CHD* coronary heart disease; *CVD* cardiovascular disease; *WHR* waist hip rate; *HRT* hormone replacement therapy**;***ADL* Activities of Daily Living; *IADLs* Instrumental Activities of Daily Living; *SBP* systolic blood pressure; *DBP* diastolic blood pressure; *HDL-CH* high density lipoprotein cholesterol; *UK* United Kingdom; *USA* United States of America; *NA* not available.

### Marital status and all-cause mortality

**Fig. 2:**
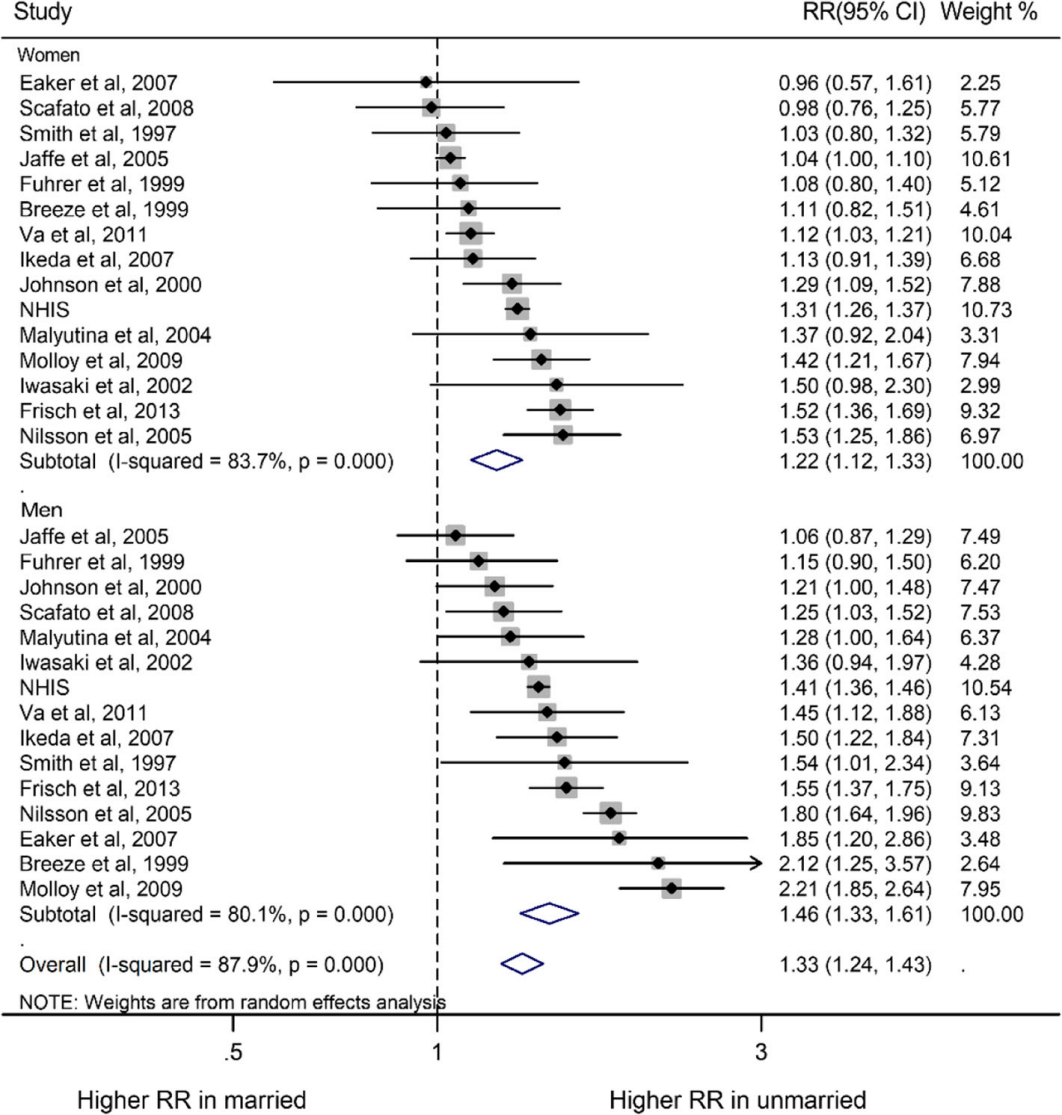
Sex-specific relative risks (RRs) for all-cause mortality, comparing non-married to married people. The boxes and lines indicate the RRs and their 95% confidence intervals (CIs) on a log scale for each study. The pooled odds ratio is represented by a diamond. The size of the gray squares indicate the relative weight of each estimate

**Fig. 3:**
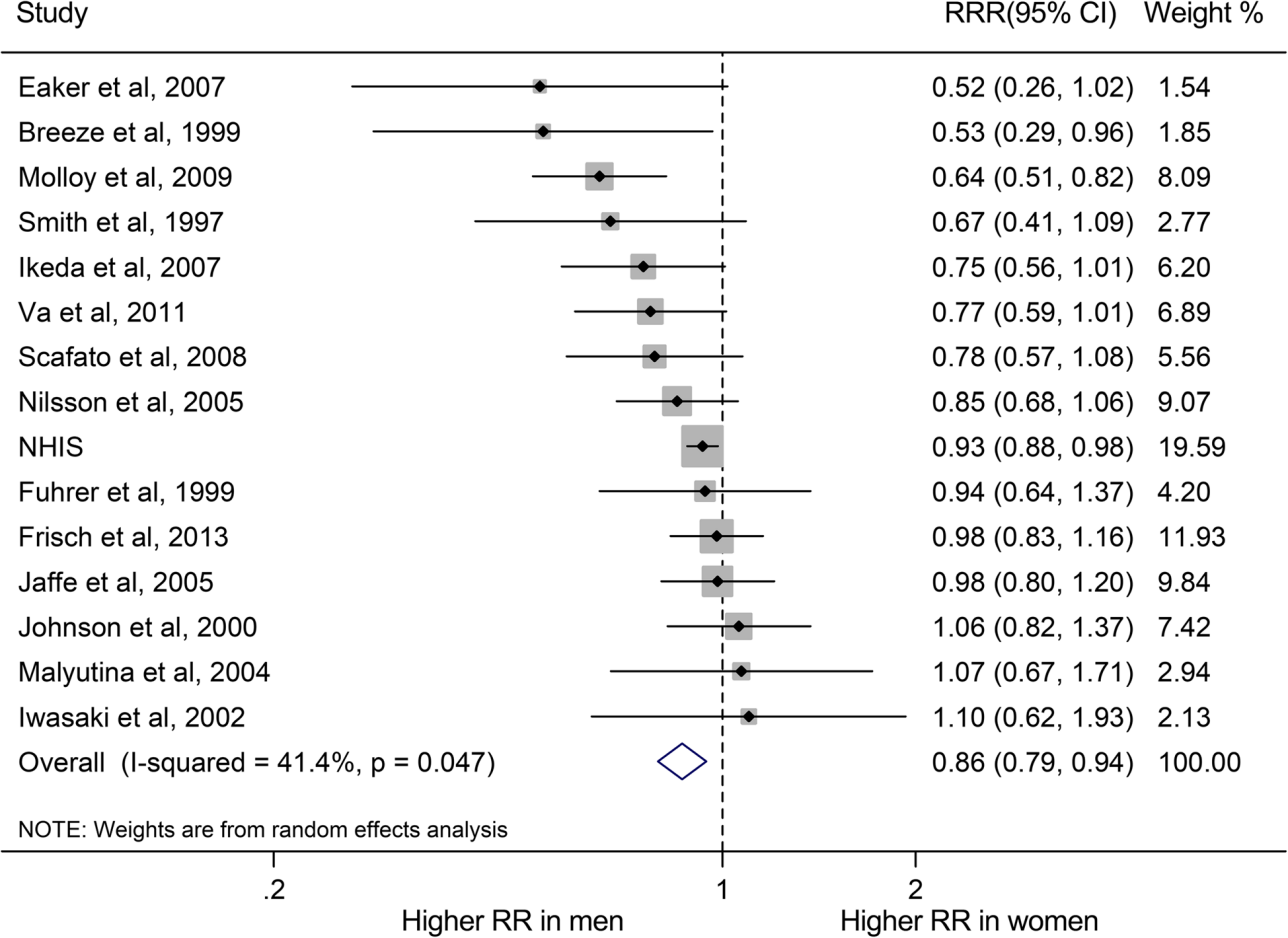
Women-to-men ratios of relative risks (RRRs) for all-cause mortality comparing non-married to married people. The boxes and lines indicate the RRRs and their 95% confidence intervals (CIs) on a log scale for each study. The pooled odds ratio is represented by a diamond. The size of the gray squares indicate the relative weight of each estimate

**Fig. 4:**
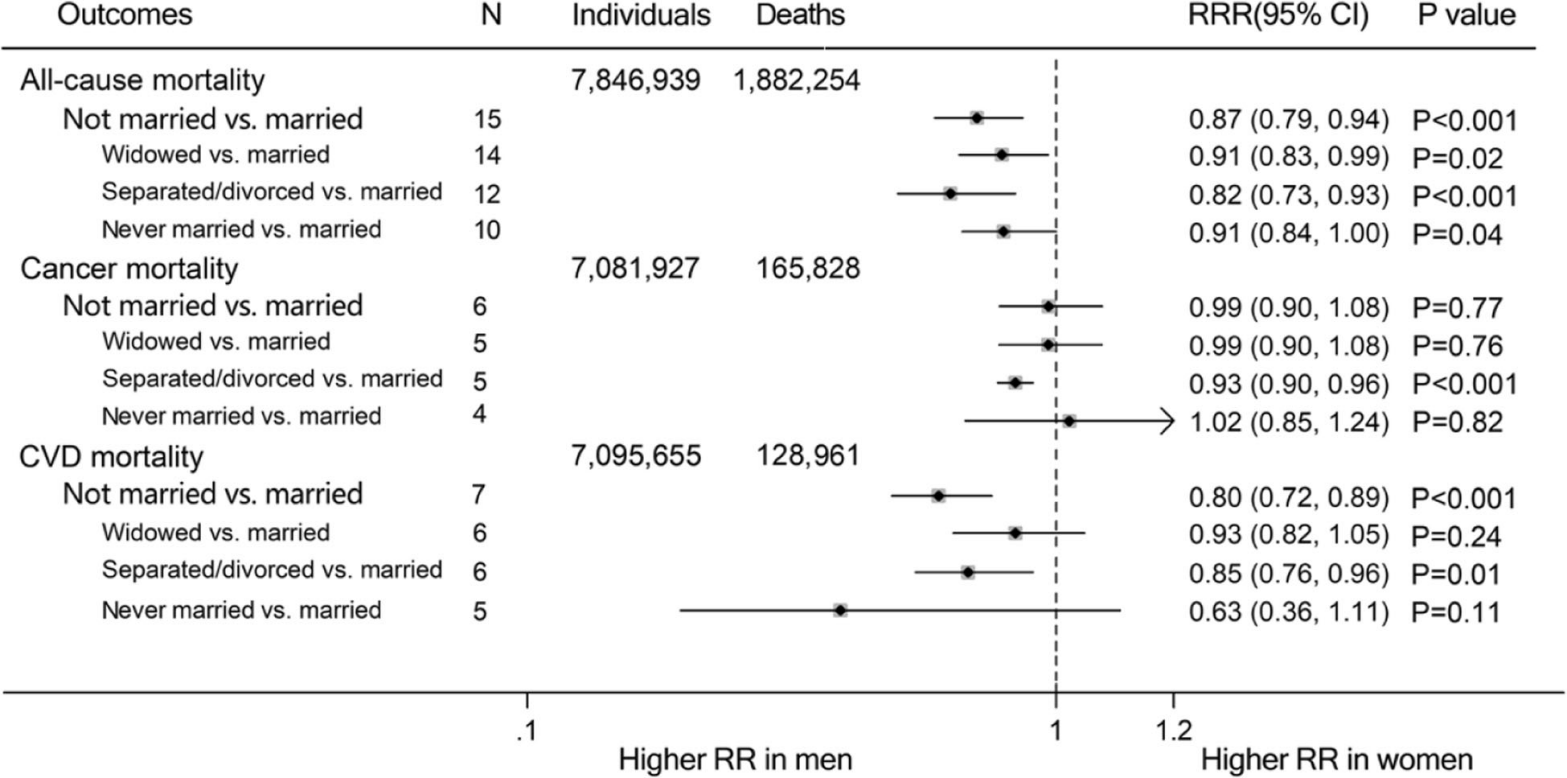
Pooled women-to-men ratios of relative risk (RRRs) for risk of all-cause, cancer and CVD mortality comparing non-married people to married people of three specific types (widowed, separated/divorced or never married). The size of the gray squares do not indicate the relative weight of each estimate

Compared with married individuals, the pooled RRs of all-cause mortality for non-married individuals were higher in both men and women (RR for men, 1.46, 95% CI, 1.33–1.61, *P* < 0.001; RR for women, 1.22, 1.12–1.33, *P* < 0.001; Fig. 2). Moreover, non-married subgroups (divorced/separated, widowed and never married) had increased risk of all-cause mortality, compared with their married people (RR for divorced/separated group: men, 1.59, 1.42–1.79; women, 1.27, 1.13–1.42; RR for widowed group: men, 1.30, 1.23–1.38; women, 1.14, 1.05–1.24; RR for never married group: men, 1.67, 1.52–1.82; women, 1.46, 1.28–1.65; Supplemental Figure 1). The pooled multiple-adjusted women-to-men RRR of risk of all-cause mortality associated with being unmarried was 0.87 (0.79–0.94; *P* = 0.001; Fig. 3; Fig. 4). Specifically, the risk of all-cause mortality for divorced/separated men was 18% higher than that for divorced/separated women (Women-to-men RRR, 0.82, 0.73–0.93, *P* < 0.001; Supplement Figure 2) while the risk of all-cause mortality were 9% higher for widowed/never married men than for widowed/never married women (Both women-to-men RRRs, 0.91, 0.84–0.99, *P* < 0.05; Supplement Figure 2).

### Marital status and cancer mortality

Compared with married men and women, unmarried men and women had 12 and 9% higher risk of cancer mortality respectively (RR for men, 1.12, 1.09–1.14, *P* < 0.001; RR for women, 1.09, 1.01–1.18, *P* = 0.03; Supplement Figure 3). Compared with married men and women, divorced/separated men and women had 16% (1.05–1.30) and 28% (1.14–1.43; Supplement Figure 3) higher risk of cancer mortality respectively. However, there was no sex difference in widowed and never married groups (All *P* > 0.05; Fig. 4). Additionally, being divorced/separated was associated with higher risk of cancer mortality in men than in women (Women-to-men RRR, 0.93, 0.90–0.96, *P* < 0.001; Supplement Figure 4). No sex differences were found between not married, widowed and never married individuals (All *P* > 0.05; Supplement Figure 4).

### Marital status and CVD mortality

**Fig. 5:**
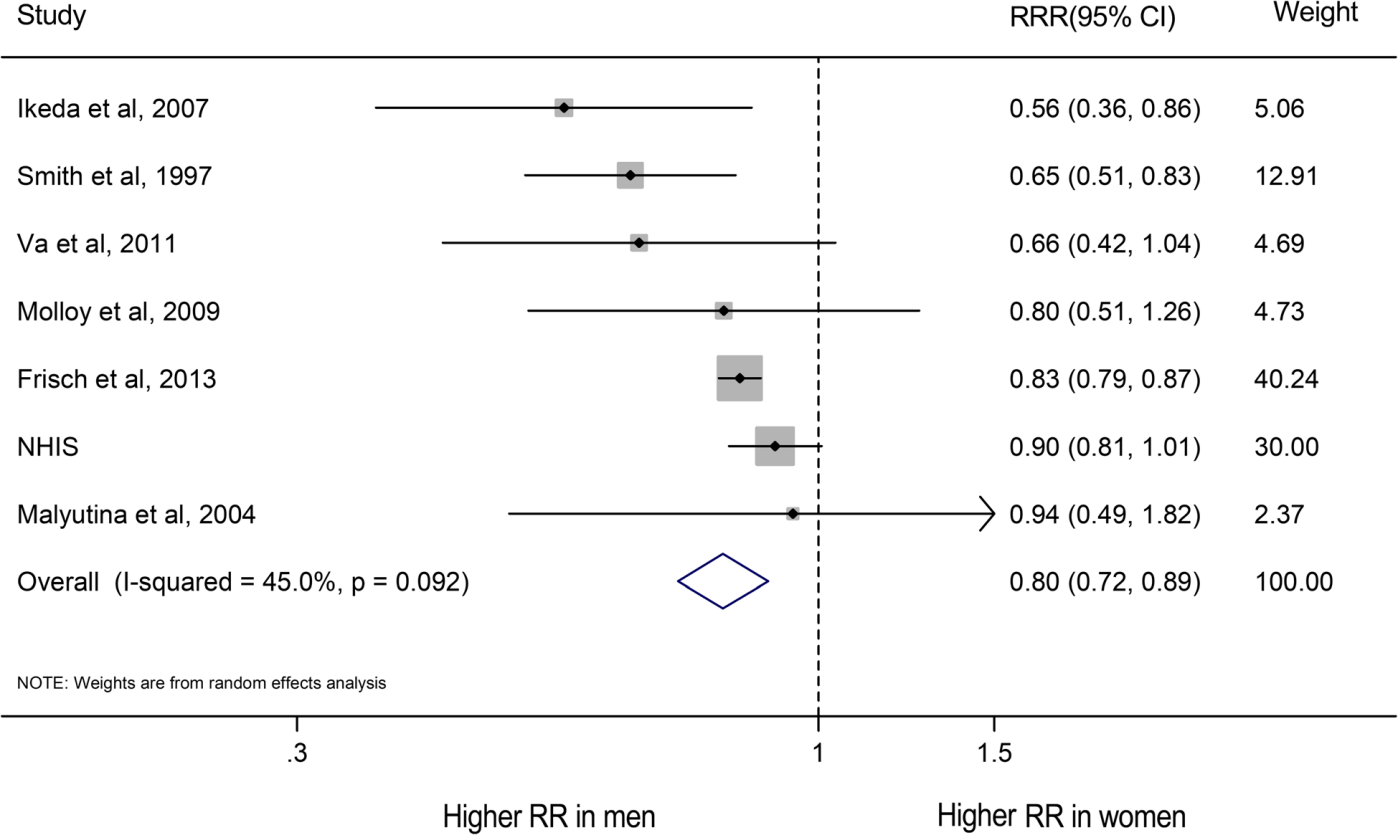
Women-to-men ratios of relative risks (RRRs) for CVD mortality comparing married to non-married people. The boxes and lines indicate the RRRs and their 95% confidence intervals (CIs) on a log scale for each study. The pooled odds ratio is represented by a diamond. The size of the gray squares indicate the relative weight of each estimate

For CVD mortality, the risk was higher in non-married participants than in married participants (RR for men, 1.60, 1.39–1.84, *P* < 0.001; RR for women, 1.19, 1.01–1.42, *P* = 0.04; Supplemental Figure 5a), regardless of being divorced/separated, widowed and never married (All *P* < 0.05, Supplement Figure 5). Likewise, compared with unmarried women, unmarried men had a 20% greater risk of CVD mortality (RRR: 0.80, 0.72–0.89, *P* < 0.001; Fig. 5). CVD mortality was greater in divorced/separated men than in divorced/separated women (women-to-men RRR: 0.85, 0.76–0.96, *P* = 0.01), but the risk is not significantly different between men and women who were widowed or never married (All *P* > 0.05, Supplement Figure 6). In addition, although a similar association was also observed in CHD and stroke mortality, there were wider CIs because their sample size is probably small (Supplement Figures 7–10). Men who never married were at a 31% excess risk of stroke when compared with women who never married (Women-to-men RRR: 0.69, 0.47–1.00, *P* = 0.05; Supplement Figure 10d).

### Meta-regression, subgroup analyses, sensitivity analyses and publication bias

For all-cause mortality, meta-regression analyses indicated that the women-to-men RRR for all-cause mortality in the widowed group decreased by 2% for every year increase in mean age (*P* = 0.003, Supplement Figure 11). It was also slightly associated with publication year and mean baseline age (P for interaction < 0.05; Supplement Figure 12; Supplement Table 3) but not correlated with study location or duration of follow-up (All *P* > 0.05). Moreover, there was no effect of the duration of follow-up, location and the publication year of studies in participants who were unmarried, divorced/separated and never married for all-cause mortality (All *P* > 0.05; Table 2). In addition, the sensitivity analyses removing each study one at a time, showed that the pooled estimates were not influenced by any single study, highlighting robustness of these findings.

**Table 2:** Sensitivity analyses of women-to-men ratio of relative risks for all-cause mortality associated with marital status

	Individuals	N	RRR	Lower	Upper	P value	Test for heterogeneity			P value for interaction
							I^2^	χ^2^	P value	
**Unmarried vs. married**	7,659,086	15								
Age (years)										0.74
<60	775,733	7	0.82	0.70	0.95	0.01	63.10%	16.24	0.01	
≥60	233,378	4	0.86	0.70	1.06	0.15	33.50%	4.51	0.21	
Others	6,649,973	4	0.94	0.82	1.07	0.34	0.00%	2.83	0.42	
Location										0.78
Asia	363,787	4	0.87	0.74	1.02	0.09	19.50%	1.7	0.29	
Europe	6,660,861	7	0.83	0.71	0.97	0.02	49.40%	2.35	0.07	
America	634,438	4	0.89	0.74	1.08	0.23	47.00%	1.2	0.13	
Others										
Follow-up years										0.30
<10	631,422	8	0.86	0.77	0.96	0.01	44.30%	12.57	0.08	
≥10	7,027,664	7	0.85	0.71	1.02	0.08	47.00%	11.32	0.08	
Publication years										0.60
≤2000	389,351	4	0.84	0.63	1.12	0.22	50.60%	6.07	0.11	
2001-2009	303,618	8	0.82	0.71	0.95	0.01	39.60%	11.59	0.12	
≥2010	6,966,117	3	0.93	0.87	0.99	0.02	7.90%	2.17	0.34	
**Widowed vs. married**	7,654,572	14								
Age (years)										0.03
<60	915,181	7	0.90	0.80	1.01	0.07	54.20%	13.11	0.04	
≥60	100,822	3	0.57	0.42	0.77	<0.001	0.00%	0.85	0.65	
Others	6,638,569	4	1.00	0.99	1.02	<0.001	0.00%	0.72	0.87	
Location										0.64
Asia	265,742	5	0.88	0.76	1.02	0.09	54.20%	5.69	0.22	
Europe	6,652,570	5	0.81	0.61	1.07	0.14	0.00%	15.24	<0.001	
America	736,260	4	0.99	0.93	1.06	0.85	0.00%	2.22	0.53	
Others	NA									
Follow-up years										0.34
<10	611,167	8	0.84	0.73	0.98	0.03	55.30%	15.66	0.03	
≥10	7,043,405	6	0.94	0.81	1.09	0.43	55.00%	11.12	0.05	
Publication years										
≤2000	375,391	2	0.71	0.36	1.39	0.31	84.10%	6.27	0.01	0.003
2001-2009	313,064	9	0.84	0.76	0.92	<0.001	0.00%	7.71	0.46	
≥2010	6,966,117	3	1.00	0.99	1.02	0.99	0.00%	0.01	1.00	
**Separated vs. married**	7,520,677	12								
Age (years)										0.60
<60	93,931	7	0.81	0.66	0.98	0.03	81.80%	32.99	<0.001	
≥60	6,649,973	1	0.57	0.18	1.76	0.33	NA	0	NA	
Others	776,773	4	0.79	0.50	1.24	0.31	69.80%	9.93	0.04	
Location										0.75
Asia	262,237	4	0.62	0.44	0.89	0.01	25.40%	4.02	0.26	
Europe	6,525,566	5	0.77	0.57	1.04	0.08	88.10%	33.71	<0.001	
America	732,874	3	0.97	0.83	1.13	0.68	61.70%	5.23	0.07	
Others	NA									
Follow-up years										0.20
<10	604,276	6	0.73	0.59	0.90	<0.001	76.70%	21.48	<0.001	
≥10	6,916,401	6	0.93	0.76	1.15	0.50	65.20%	14.36	0.01	
Publication years										0.94
≤2000	375,391	2	1.01	0.65	1.57	0.96	23.00%	1.3	0.26	
2001-2009	179,169	7	0.72	0.59	0.87	<0.001	34.20%	9.12	0.17	
≥2010	6,966,117	3	0.89	0.80	1.00	0.05	80.20%	10.12	0.01	
**Never married vs. married**	7,511,483	10								
Age (years)										0.20
<60	767,579	5	0.89	0.81	0.97	0.01	22.40%	5.16	0.3	
≥60	93,931	1	0.54	0.31	0.95	0.03	NA	0	NA	
Others	6,649,973	4	0.99	0.97	1.02	0.59	0.00%	0.95	0.8	
Location										0.51
Asia	232,631	3	0.87	0.66	1.15	0.31	0.00%	1.62	0.44	
Europe	6,658,279	5	0.85	0.71	1.01	0.06	71.40%	14.01	0.01	
America	620,573	2	0.94	0.86	1.03	0.18	0.00%	0.62	0.43	
Others	NA									
Follow-up years										0.47
<10	447,064	3	0.93	0.83	1.04	0.07	40.20%	3.34	0.19	
≥10	7,052,854	6	0.84	0.70	1.02	0.21	50.20%	10.04	0.07	
Others	11,565	1	1.44	0.59	3.50	0.42	NA	0	NA	
Publication years										0.21
≤2000	414,446	3	0.86	0.69	1.08	0.19	58.20%	4.78	0.09	
2001-2009	130,920	4	0.77	0.61	0.98	0.03	0.00%	2.55	0.59	
≥2010	6,966,117	3	0.99	0.97	1.01	0.38	0.00%	1.95	0.38	

Abbreviations: *N* number of studies; *NA* not available.

Publication bias was found for CHD mortality in divorced/separated group (Egger’s test *P* = 0.03) and for all-cause mortality in widowed and never married groups (Both *P* = 0.003; Supplement Figure 13); however, the trim-and-fill analysis did not change the overall results, and there was no evidence of publication bias for other endpoints (All *P* > 0.05).

## Discussion

This meta-analysis, which included data of more than 7,000,000 men and women, indicated that compared with being married, being unmarried was associated with higher risk of all-cause mortality, cancer mortality, CVD mortality and CHD mortality. This was especially true for those who had never been married regardless of their gender. However, the association with death from all-cause and CVD was stronger in men. Compared with women who were divorced or separated, men had higher risk of all-cause mortality, cancer mortality and CVD mortality after the dissolution of marriage. Moreover, men who never married were at 31 and 9% separately higher excess risk of stroke mortality and all-cause mortality compared with never married women, but not CHD mortality.

Marital status appears to be a critical factor of mortality outcomes across different countries and cultures [23, 24]. A recent meta-analysis also showed that being unmarried was associated with increased risk of CHD death or stroke death in both men and women compared with married participants [19]. Compared with married people, unmarried individuals may obtain less emotional, financial and companionship support and can even experience more sub-clinical symptoms of depression and anxiety [49–51], and major mental disorder [52]. In addition, marriage selection theory proposes that healthier individuals were more likely to marry or stay married because of the physical and psychological advantageous attributes [53]. This may help explain why unmarried people had higher mortality than married people in the present study.

Our results showed that being unmarried is particularly more dangerous for men than for women with respect to CVD and all-cause mortality. This is consistent with findings from the previous meta-analysis which indicated that men who were single generally had the poorest health outcomes of any type among all unmarried conditions [54]. The potential mechanisms for such findings are likely to be biological, psychological and social in nature. From a biological standpoint, acute stressors which trigger activities of Hypothalamic-Pituitary-Adrenal (HPA) axis and sympathetic nervous system [8, 35] and result in output of stress hormones such as cortisol, have been found to be pronounced in men when compared to women [55, 56]. Increased cortisol production has been linked to higher rate of morbidity and poorer health outcomes [8]. In addition, HPA axis can mediate the production of sex hormones [57]. In women, estrogen can protect women against heart disease by reducing circulatory levels of harmful cholesterol [58], whereas testosterone increased the concentrations of low-density lipoprotein and inflammatory markers that can drive the progression of atherosclerosis and stroke [59–61] in men. Further, there is evidence that women have stronger immune systems, in part because testosterone caused immunosuppression and more frequently infection [60, 62].

From a psychological perspective, women who provide more social support to others and are more engaged in their social networks are shown to be buffered or at least better equipped to deal with stress. The New England Research Institute reported that 66% of men rely on their wives for their primary social supports [63]. Men living alone are more likely to disregard professional’s advice [64], have smaller and less intimate social networks, are more likely to be lonely and suffer depression than women with similar partner histories [65, 66].

From a social behavioral perspective, the social conditioning process may contribute to the influence on marital status in male’s increased risk of mortality. Indeed, married men fare better than those who have never been or were previously married. In most Western cultures, boys and young men are often conditioned to feel they are responsible for providing financially for a family. A lack of adherence to this gender norm may result in feelings of perceived hopelessness or inadequacy that impact physical health. For those men who were previously married, there is some evidence that the association between marital status and mortality is largely explained by the length of marriage and early life history such as childhood disadvantage [25]. Taken together, this suggests that a life course approach is required to understand the link between marriage and mortality risk.

In comparison with most men who had a more sedentary lifestyle [67–70], adult women under the age of 65 who were reported more doctor visits and go to gym more often than men, with the gender difference widest among individuals aged 18–44 [71]. Moreover, men who drink more alcohol and more smoking than women [72] were 4.5 times as likely to die from CHD in middle age [73] and twice from cirrhosis [74, 75] and more vulnerable to respiratory illnesses, such as COPD and lung cancer, and had more excess risk of death from these diseases.

Our results provide a social context in which to consider why sex-specific differences in individual level risk factors may exist. Recently, traditional CVD-related risk factors, such as smoking and diabetes have been demonstrated to confer greater excess risk of CHD and stroke for women than men. For example, both American Heart Association guidelines and European Society of Cardiology guidelines recommended that women with diabetes should exercise more to counteract the higher excess risk of CVD conferred by diabetes in women than that in men [76–78]. If women do not appear to benefit from marriage to the extent of their male counterparts, this needs to be considered. Low marital quality appears to be linked to women’s health behaviors and disease markers including low HDL cholesterol, high triglycerides, and higher BMI, blood pressure and is also a risk factor for recurrent heart attack [79]. Expectations of women as mothers and wives as they relate to caregiving and parenting places them a higher risk of non-fatal CHD in middle age [80]. Our findings that men yield greater mortality risk owing to the absence of marriage support the idea that they have more to lose from marriage dissolution or from never marrying when compared with women who do not attract such a mortality benefit.

### Strengths and limitations

Strengths of this study included the use of a large sample to evaluate sex difference between marital status and cause-specific outcomes. This is advantageous as it can minimize the role of confounding factors. However, several of these issues remain. The meta-analysis was based on prospective cohort studies, therefore, the conventional problems of confounding effects and potential bias in observational study were inevitable. Although our study had a large sample size and for each study we used the estimates from the multiple-adjusted models, which could reduce the confounding and bias, the possible influence of other risk factors could not be ruled out and we are unable to determine causation. Secondly, although we were not able to adjust properly for baseline differences in confounding factors both between and within studies which may explain the small difference observed in our meta-analysis, the sensitivity and subgroup analyses were used to assess the disparities in all the subgroups, and moreover the meta-regression was also performed to evaluate and reduce the heterogeneity among the studies. Thirdly, further sub-categories of marriage or intimate partnerships remained unexamined in this study. For example, marriages with high levels of dissatisfaction and/or conflict may produce poor health outcomes (as has been shown for women especially); long term partnerships that are not officially defined under the traditional definition of marriage may confer positive health benefits particularly through long term cohabitation; the role of children in the marriage and their effect of health requires further exploration. Fourthly, there was an evidence of publication bias for all-cause mortality in widowed and divorced/separated groups, for CVD mortality in never married group and stroke mortality in widowed group. Although the use of trim and fill procedures did not result in the change of the results of our meta-analysis, the possibility of an artifact of unpublished negative studies could not be ignored with this method. Fifthly, these included studies only involved papers published in English language, although publication bias was analyzed, lack of papers published in other language except English limited more in-depth analyses than were reported here.

## Conclusion

Unmarried men (divorced, widowed or never married) have excess risk of stroke mortality and all-cause mortality compared to women. Moreover, men whose marriages were dissolved had higher risk of both cancer and CVD mortality. Compared to their single female counterparts, single men were at higher risk of stroke mortality. Our results also warrant consideration as to why women do not appear to benefit from marriage to the same extent as men. Further studies are warranted to clarify the biological, behavioral, or social mechanisms that may drive these associations in order to make conclusions about its application to public health policy and allocation of public health resources.

## Supplementary information

**Additional file 1 Supplemental Table 1.** PRISMA 2009 Checklist. **Supplemental Table 2.** Quality of included studies assessed with Newcastle-Ottawa Scale. **Supplemental Table 3.** Subgroup analyses of women-to-men ratio of relative risks for all-cause mortality associated with marital status. **Supplemental Figure 1.** Sex-specific relative risks (RRs) for all-cause mortality, comparing widowed, divorced/separated and never married to married people: (a) Sex-specific RRs for all-cause mortality, comparing widowed to married people; (b) Sex-specific RRs for all-cause mortality, comparing divorced/separated to married people; (c) Sex-specific RRs for all-cause mortality, comparing never married to married people. **Supplemental Figure 2.** Women-to-men ratios of relative risks (RRRs) for all-cause mortality comparing widowed, divorced/separated and never married to married people: (a) Women-to-men RRRs for all-cause mortality comparing widowed to married people; (b) Women-to-men RRRs for all-cause mortality comparing divorced/separated to married people; (c) Women-to-men RRRs for all-cause mortality comparing never married to married people. **Supplemental Figure 3.** Sex-specific relative risks (RRs) for cancer mortality, comparing non-married, widowed, divorced/separated and never married to married people: (a) Sex-specific RRs for cancer mortality, comparing non-married to married people; (b) Sex-specific RRs for cancer mortality, comparing widowed to married people; (c) Sex-specific RRs for cancer mortality, comparing divorced/separated to married people; (d) Sex-specific RRs for cancer mortality, comparing never married to married people. **Supplemental Figure 4.** Women-to-men ratios of relative risks (RRRs) for cancer mortality comparing non-married, widowed, divorced/separated and never married to married people: (a) Women-to-men RRRs for cancer mortality comparing non-married to married people; (b) Women-to-men RRRs for cancer mortality comparing widowed to married people; (c) Women-to-men RRRs for cancer mortality comparing divorced/separated to married people; (d) Women-to-men RRRs for cancer mortality comparing never married to married people. **Supplemental Figure 5.** Sex-specific relative risks (RRs) for cardiovascular (CV) mortality, comparing non-married, widowed, divorced/separated and never married to married people: (a) Sex-specific RRs for CV mortality, comparing non-married to married people; (b) Sex-specific RRs for CV mortality, comparing widowed to married people; (c) Sex-specific RRs for CV mortality, comparing divorced/separated to married people; (d) Sex-specific RRs for CV mortality, comparing never married to married people. **Supplemental Figure 6.** Women-to-men ratios of relative risks (RRRs) for cardiovascular (CV) mortality comparing non-married, widowed, divorced/separated and never married to married people: (a) Women-to-men RRRs for CV mortality comparing non-married to married people; (b) Women-to-men RRRs for CV mortality comparing widowed to married people; (c) Women-to-men RRRs for CV mortality comparing divorced/separated to married people; (d) Women-to-men RRRs for CV mortality comparing never married to married people. **Supplemental Figure 7.** Sex-specific relative risks (RRs) for coronary heart disease (CHD) mortality, comparing non-married, widowed, divorced/separated and never married to married people: (a) Sex-specific RRs for CHD mortality, comparing non-married to married people; (b) Sex-specific RRs for CHD mortality, comparing widowed to married people; (c) Sex-specific RRs for CHD mortality, comparing divorced/separated to married people; (d) Sex-specific RRs for CHD mortality, comparing never married to married people. **Supplemental Figure 8.** Women-to-men ratios of relative risks (RRRs) for coronary heart disease (CHD) mortality comparing non-married, widowed, divorced/separated and never married to married people: (a) Women-to-men RRRs for CHD mortality comparing non-married to married people; (b) Women-to-men RRRs for CHD mortality comparing widowed to married people; (c) Women-to-men RRRs for CHD mortality comparing divorced/separated to married people; (d) Women-to-men RRRs for CV mortality comparing never married to married people. **Supplemental Figure 9.** Sex-specific relative risks (RRs) for stroke mortality, comparing non-married, widowed, divorced/separated and never married to married people: (a) Sex-specific RRs for stroke mortality, comparing non-married to married people; (b) Sex-specific RRs for stroke mortality, comparing widowed to married people; (c) Sex-specific RRs for stroke mortality, comparing divorced/separated to married people; (d) Sex-specific RRs for stroke mortality, comparing never married to married people. **Supplemental Figure 10.** Women-to-men ratios of relative risks (RRRs) for stroke mortality comparing non-married, widowed, divorced/separated and never married to married people: (a) Women-to-men RRRs for stroke mortality comparing non-married to married people; (b) Women-to-men RRRs for stroke mortality comparing widowed to married people; (c) Women-to-men RRRs for stroke mortality comparing divorced/separated to married people; (d) Women-to-men RRRs for stroke mortality comparing never married to married people. **Supplemental Figure 11.** Meta-regression for mean age at baseline for pooled women-to-men ratios of relative risk for risk of all-cause mortality comparing non-married people to married people. **Supplemental Figure 12.** Subgroup analyses for all-cause mortality for pooled women-to-men ratios of relative risk (RRRs) for risk of all-cause mortality comparing widowed people to married people. **Supplemental Figure 13.** Begg’s publication bias plot for the pooled ratio of women-to-men relative risks: (a) Begg’s publication bias plot for CHD mortality in divorced/separated group; (b) Begg’s publication bias plot for all-cause mortality in widowed group; (c) Begg’s publication bias plot for all-cause mortality in never married group; Abbreviations: CHD: coronary heart disease; RR: relative risk; RRR: ratio of RR.

## Data Availability

The following information was supplied regarding data availability: This is a systematic review of the literature. No raw data was analysed.
