# Supplementary material for: Sex differences in the association between marital status and the risk of cardiovascular, cancer, and all-cause mortality: a systematic review and meta-analysis of 7,881,040 individuals
Source: Glob Health Res Policy. 2020 Feb 28;5:4. doi: 10.1186/s41256-020-00133-8 (PMC7047380; doi:10.1186/s41256-020-00133-8)
Supplement: Supplementary file 1 — Additional file 1 Supplemental Table 1. PRISMA 2009 Checklist. Supplemental Table 2. Quality of included studies assessed with Newcastle-Ottawa Scale. Supplemental Table 3. Subgroup analyses of women-to-men ratio of relative risks for all-cause mortality associated with marital status. Supplemental Figure 1. Sex-specific relative risks (RRs) for all-cause mortality, comparing widowed, divorced/separated and never married to married people: (a) Sex-specific RRs for all-cause mortality, comparing widowed to married people; (b) Sex-specific RRs for all-cause mortality, comparing divorced/separated to married people; (c) Sex-specific RRs for all-cause mortality, comparing never married to married people. Supplemental Figure 2. Women-to-men ratios of relative risks (RRRs) for all-cause mortality comparing widowed, divorced/separated and never married to married people: (a) Women-to-men RRRs for all-cause mortality comparing widowed to married people; (b) Women-to-men RRRs for all-cause mortality comparing divorced/separated to married people; (c) Women-to-men RRRs for all-cause mortality comparing never married to married people. Supplemental Figure 3. Sex-specific relative risks (RRs) for cancer mortality, comparing non-married, widowed, divorced/separated and never married to married people: (a) Sex-specific RRs for cancer mortality, comparing non-married to married people; (b) Sex-specific RRs for cancer mortality, comparing widowed to married people; (c) Sex-specific RRs for cancer mortality, comparing divorced/separated to married people; (d) Sex-specific RRs for cancer mortality, comparing never married to married people. Supplemental Figure 4. Women-to-men ratios of relative risks (RRRs) for cancer mortality comparing non-married, widowed, divorced/separated and never married to married people: (a) Women-to-men RRRs for cancer mortality comparing non-married to married people; (b) Women-to-men RRRs for cancer mortality comparing widowed to married people; (c) Wome [file 41256_2020_133_MOESM1_ESM.docx]

**Sex differences in the relationship between marital status and risk of cardiovascular disease, cancer, and all-cause mortality: a systematic review and meta-analysis of prospective cohort studies**

**Online Supplemental Material**

The following materials are included in the Online Supplemental Material.

1. Search strategies in PubMed and EMBASE.

2. Supplemental Tables 1-5.

3. Supplemental Figures 1-11.

**Search strategies in PubMed**

#1 Search marriage [MeSH Terms] OR marital status[MeSH Terms] OR divorce[MeSH Terms] OR Widowed[MeSH Terms] OR Separated[MeSH Terms]

#2 Search all cause mortality[MeSH Terms] OR all-cause mortality[MeSH Terms] OR total mortality[MeSH Terms] OR cardiovascular disease[MeSH Terms] OR coronary artery disease[MeSH Terms] OR coronary heart disease[MeSH Terms] OR ischemic heart disease[MeSH Terms] OR cerebrovascular disorder[MeSH Terms] OR stroke[MeSH Terms] OR neoplasm[MeSH Terms] OR cancer[MeSH Terms]

#3 Search mortality[MeSH Terms] OR death[MeSH Terms]

#4 Search epidemiologic studies[MeSH Terms] OR epidemiologic[MeSH Terms] OR cohort[MeSH Terms] OR follow [MeSH Terms] OR prospective[MeSH Terms]

#5 #1 AND #2 AND #3 AND #4 Filters: Humans

**Search strategies in Embase**

#1 'marriage'

#2 'mortality' OR 'cause of death' OR 'cardiovascular disease' OR 'malignant neoplasm'

#3 #1 AND #2

#4 #3 AND ('cohort analysis'/de OR 'longitudinal study'/de OR 'observational study'/de OR 'prospective study'/de)

**Supplemental Table 1:** **PRISMA 2009 Checklist**

| **Section/topic** | **#** | **Checklist item** | **Reported on page #** |
| --- | --- | --- | --- |
| **TITLE** | | |  |
| Title | 1 | Identify the report as a systematic review, meta-analysis, or both. | 1 |
| **ABSTRACT** | | |  |
| Structured summary | 2 | Provide a structured summary including, as applicable: background; objectives; data sources; study eligibility criteria, participants, and interventions; study appraisal and synthesis methods; results; limitations; conclusions and implications of key findings; systematic review registration number. | 2 |
| **INTRODUCTION** | | |  |
| Rationale | 3 | Describe the rationale for the review in the context of what is already known. | 3 |
| Objectives | 4 | Provide an explicit statement of questions being addressed with reference to participants, interventions, comparisons, outcomes, and study design (PICOS). | 3,4 |
| **METHODS** | | |  |
| Protocol and registration | 5 | Indicate if a review protocol exists, if and where it can be accessed (e.g., Web address), and, if available, provide registration information including registration number. | 4 |
| Eligibility criteria | 6 | Specify study characteristics (e.g., PICOS, length of follow-up) and report characteristics (e.g., years considered, language, publication status) used as criteria for eligibility, giving rationale. | 4,5 |
| Information sources | 7 | Describe all information sources (e.g., databases with dates of coverage, contact with study authors to identify additional studies) in the search and date last searched. | 5 |
| Search | 8 | Present full electronic search strategy for at least one database, including any limits used, such that it could be repeated. | 4,5 |
| Study selection | 9 | State the process for selecting studies (i.e., screening, eligibility, included in systematic review, and, if applicable, included in the meta-analysis). | 5 |
| Data collection process | 10 | Describe method of data extraction from reports (e.g., piloted forms, independently, in duplicate) and any processes for obtaining and confirming data from investigators. | 5 |
| Data items | 11 | List and define all variables for which data were sought (e.g., PICOS, funding sources) and any assumptions and simplifications made. | 5 |
| Risk of bias in individual studies | 12 | Describe methods used for assessing risk of bias of individual studies (including specification of whether this was done at the study or outcome level), and how this information is to be used in any data synthesis. | 5 |
| Summary measures | 13 | State the principal summary measures (e.g., risk ratio, difference in means). | 5 |
| Synthesis of results | 14 | Describe the methods of handling data and combining results of studies, if done, including measures of consistency (e.g., I^2^) for each meta-analysis. | 5,6 |
| Risk of bias across studies | 15 | Specify any assessment of risk of bias that may affect the cumulative evidence (e.g., publication bias, selective reporting within studies). | 6 |
| Additional analyses | 16 | Describe methods of additional analyses (e.g., sensitivity or subgroup analyses, meta-regression), if done, indicating which were pre-specified. | 6 |
| **RESULTS** | | |  |
| Study selection | 17 | Give numbers of studies screened, assessed for eligibility, and included in the review, with reasons for exclusions at each stage, ideally with a flow diagram. | 7 |
| Study characteristics | 18 | For each study, present characteristics for which data were extracted (e.g., study size, PICOS, follow-up period) and provide the citations. | 7 |
| Risk of bias within studies | 19 | Present data on risk of bias of each study and, if available, any outcome level assessment (see item 12). | 7 |
| Results of individual studies | 20 | For all outcomes considered (benefits or harms), present, for each study: (a) simple summary data for each intervention group (b) effect estimates and confidence intervals, ideally with a forest plot. | 7-9 |
| Synthesis of results | 21 | Present results of each meta-analysis done, including confidence intervals and measures of consistency. | 7-9 |
| Risk of bias across studies | 22 | Present results of any assessment of risk of bias across studies (see Item 15). | 7-9 |
| Additional analysis | 23 | Give results of additional analyses, if done (e.g., sensitivity or subgroup analyses, meta-regression [see Item 16]). | 7-10 |
| **DISCUSSION** | | |  |
| Summary of evidence | 24 | Summarize the main findings including the strength of evidence for each main outcome; consider their relevance to key groups (e.g., healthcare providers, users, and policy makers). | 10-13 |
| Limitations | 25 | Discuss limitations at study and outcome level (e.g., risk of bias), and at review-level (e.g., incomplete retrieval of identified research, reporting bias). | 13 |
| Conclusions | 26 | Provide a general interpretation of the results in the context of other evidence, and implications for future research. | 13 |
| **FUNDING** | | |  |
| Funding | 27 | Describe sources of funding for the systematic review and other support (e.g., supply of data); role of funders for the systematic review. | 1 |

**Supplemental Table 2. Quality of included studies assessed with Newcastle-Ottawa Scale**

| **Author** | **Selection** | | | | **Comparability** | **Outcome** | | | **Overall quality** |
| --- | --- | --- | --- | --- | --- | --- | --- | --- | --- |
|  | **Representative of the exposed cohort** | **Selection of the non-exposed cohort** | **Exposure ascertainment** | **Demonstration that outcome of interest was not present at start of study** | **Comparability of cohorts** | **Outcome assessment** | **Adequate follow-up time (> 5 years)** | **Adequacy of follow up of cohorts** |  |
| Va et al, 2011^27^ | 1 | 1 | 1 | 1 | 2 | 1 | 0 | 1 | 8 |
| Frisch et al, 2013^28^ | 1 | 1 | 1 | 1 | 2 | 1 | 1 | 1 | 9 |
| Molloy et al, 2009^30^ | 1 | 1 | 1 | 1 | 1 | 1 | 1 | 1 | 8 |
| Dupre et al, 2009^31^ | 1 | 1 | 1 | 1 | 2 | 1 | 1 | 1 | 9 |
| Scafato et al, 2006^32^ | 1 | 1 | 1 | 1 | 2 | 1 | 1 | 1 | 9 |
| Stimpson et al, 2008^33^ | 1 | 1 | 1 | 1 | 2 | 1 | 1 | 1 | 9 |
| Ikeda et al, 2007^34^ | 1 | 1 | 0 | 1 | 2 | 1 | 1 | 1 | 8 |
| Eaker et al, 2007^36^ | 1 | 1 | 0 | 1 | 2 | 0 | 0 | 1 | 6 |
| Jaffe et al, 2005^37^ | 1 | 1 | 1 | 1 | 2 | 1 | 1 | 1 | 9 |
| Nilsson et al, 2005^38^ | 1 | 1 | 0 | 1 | 2 | 1 | 1 | 1 | 8 |
| Hurt et al, 2003^39^ | 1 | 1 | 1 | 1 | 2 | 1 | 1 | 0 | 8 |
| Strand et al, 2004^40^ | 1 | 1 | 0 | 1 | 2 | 1 | 1 | 0 | 7 |
| Malyutina et al, 2004^41^ | 1 | 1 | 1 | 1 | 2 | 1 | 1 | 0 | 8 |
| Nagata et al, 2003^42^ | 1 | 1 | 0 | 1 | 2 | 1 | 1 | 0 | 7 |
| Johnson et al, 2000^43^ | 1 | 1 | 1 | 1 | 2 | 1 | 1 | 1 | 9 |
| Iwasaki et al, 2002^44^ | 1 | 1 | 0 | 1 | 2 | 0 | 1 | 0 | 6 |
| Breeze et al, 1999^45^ | 1 | 1 | 1 | 1 | 2 | 1 | 1 | 0 | 8 |
| Fuhrer et al, 1999^46^ | 1 | 1 | 1 | 1 | 2 | 1 | 0 | 0 | 7 |
| Smith et al, 1997^35^ | 1 | 1 | 1 | 1 | 2 | 1 | 1 | 0 | 8 |
| Nilsson et al, 1998^29^ | 1 | 1 | 1 | 1 | 2 | 1 | 1 | 1 | 9 |
| NHIS, 2018 | 1 | 1 | 1 | 1 | 2 | 1 | 1 | 1 | 9 |

**Supplemental Table 3. Subgroup analyses of women-to-men ratio of relative risks for all-cause mortality associated with marital status.**

|  | Individuals | N | RRR | Lower | Upper | P value | Test for heterogeneity | | | P value for interaction |
| --- | --- | --- | --- | --- | --- | --- | --- | --- | --- | --- |
|  |  |  |  |  |  |  | I^2^ | χ^2^ | P value |  |
| **Unmarried vs. married** | 7,659,086 | 15 |  |  |  |  |  |  |  |  |
| Age (years) |  |  |  |  |  |  |  |  |  | 0.74 |
| <60 | 775,733 | 7 | 0.82 | 0.70 | 0.95 | 0.01 | 63.10% | 16.24 | 0.01 |  |
| ≥60 | 233,378 | 4 | 0.86 | 0.70 | 1.06 | 0.15 | 33.50% | 4.51 | 0.21 |  |
| Others | 6,649,973 | 4 | 0.94 | 0.82 | 1.07 | 0.34 | 0.00% | 2.83 | 0.42 |  |
| Location |  |  |  |  |  |  |  |  |  | 0.78 |
| Asia | 363,787 | 4 | 0.87 | 0.74 | 1.02 | 0.09 | 19.50% | 1.7 | 0.29 |  |
| Europe | 6,660,861 | 7 | 0.83 | 0.71 | 0.97 | 0.02 | 49.40% | 2.35 | 0.07 |  |
| America | 634,438 | 4 | 0.89 | 0.74 | 1.08 | 0.23 | 47.00% | 1.2 | 0.13 |  |
| Others |  |  |  |  |  |  |  |  |  |  |
| Follow-up years |  |  |  |  |  |  |  |  |  | 0.30 |
| <10 | 631,422 | 8 | 0.86 | 0.77 | 0.96 | 0.01 | 44.30% | 12.57 | 0.08 |  |
| ≥10 | 7,027,664 | 7 | 0.85 | 0.71 | 1.02 | 0.08 | 47.00% | 11.32 | 0.08 |  |
| Publication years |  |  |  |  |  |  |  |  |  | 0.60 |
| ≤2000 | 389,351 | 4 | 0.84 | 0.63 | 1.12 | 0.22 | 50.60% | 6.07 | 0.11 |  |
| 2001-2009 | 303,618 | 8 | 0.82 | 0.71 | 0.95 | 0.01 | 39.60% | 11.59 | 0.12 |  |
| ≥2010 | 6,966,117 | 3 | 0.93 | 0.87 | 0.99 | 0.02 | 7.90% | 2.17 | 0.34 |  |
| **Widowed vs. married** | 7,654,572 | 14 |  |  |  |  |  |  |  |  |
| Age (years) |  |  |  |  |  |  |  |  |  | 0.03 |
| <60 | 915,181 | 7 | 0.90 | 0.80 | 1.01 | 0.07 | 54.20% | 13.11 | 0.04 |  |
| ≥60 | 100,822 | 3 | 0.57 | 0.42 | 0.77 | <0.001 | 0.00% | 0.85 | 0.65 |  |
| Others | 6,638,569 | 4 | 1.00 | 0.99 | 1.02 | <0.001 | 0.00% | 0.72 | 0.87 |  |
| Location |  |  |  |  |  |  |  |  |  | 0.64 |
| Asia | 265,742 | 5 | 0.88 | 0.76 | 1.02 | 0.09 | 54.20% | 5.69 | 0.22 |  |
| Europe | 6,652,570 | 5 | 0.81 | 0.61 | 1.07 | 0.14 | 0.00% | 15.24 | <0.001 |  |
| America | 736,260 | 4 | 0.99 | 0.93 | 1.06 | 0.85 | 0.00% | 2.22 | 0.53 |  |
| Others | NA |  |  |  |  |  |  |  |  |  |
| Follow-up years |  |  |  |  |  |  |  |  |  | 0.34 |
| <10 | 611,167 | 8 | 0.84 | 0.73 | 0.98 | 0.03 | 55.30% | 15.66 | 0.03 |  |
| ≥10 | 7,043,405 | 6 | 0.94 | 0.81 | 1.09 | 0.43 | 55.00% | 11.12 | 0.05 |  |
| Publication years |  |  |  |  |  |  |  |  |  |  |
| ≤2000 | 375,391 | 2 | 0.71 | 0.36 | 1.39 | 0.31 | 84.10% | 6.27 | 0.01 | 0.003 |
| 2001-2009 | 313,064 | 9 | 0.84 | 0.76 | 0.92 | <0.001 | 0.00% | 7.71 | 0.46 |  |
| ≥2010 | 6,966,117 | 3 | 1.00 | 0.99 | 1.02 | 0.99 | 0.00% | 0.01 | 1.00 |  |
| **Separated vs. married** | 7,520,677 | 12 |  |  |  |  |  |  |  |  |
| Age (years) |  |  |  |  |  |  |  |  |  | 0.60 |
| <60 | 93,931 | 7 | 0.81 | 0.66 | 0.98 | 0.03 | 81.80% | 32.99 | <0.001 |  |
| ≥60 | 6,649,973 | 1 | 0.57 | 0.18 | 1.76 | 0.33 | NA | 0 | NA |  |
| Others | 776,773 | 4 | 0.79 | 0.50 | 1.24 | 0.31 | 69.80% | 9.93 | 0.04 |  |
| Location |  |  |  |  |  |  |  |  |  | 0.75 |
| Asia | 262,237 | 4 | 0.62 | 0.44 | 0.89 | 0.01 | 25.40% | 4.02 | 0.26 |  |
| Europe | 6,525,566 | 5 | 0.77 | 0.57 | 1.04 | 0.08 | 88.10% | 33.71 | <0.001 |  |
| America | 732,874 | 3 | 0.97 | 0.83 | 1.13 | 0.68 | 61.70% | 5.23 | 0.07 |  |
| Others | NA |  |  |  |  |  |  |  |  |  |
| Follow-up years |  |  |  |  |  |  |  |  |  | 0.20 |
| <10 | 604,276 | 6 | 0.73 | 0.59 | 0.90 | <0.001 | 76.70% | 21.48 | <0.001 |  |
| ≥10 | 6,916,401 | 6 | 0.93 | 0.76 | 1.15 | 0.50 | 65.20% | 14.36 | 0.01 |  |
| Publication years |  |  |  |  |  |  |  |  |  | 0.94 |
| ≤2000 | 375,391 | 2 | 1.01 | 0.65 | 1.57 | 0.96 | 23.00% | 1.3 | 0.26 |  |
| 2001-2009 | 179,169 | 7 | 0.72 | 0.59 | 0.87 | <0.001 | 34.20% | 9.12 | 0.17 |  |
| ≥2010 | 6,966,117 | 3 | 0.89 | 0.80 | 1.00 | 0.05 | 80.20% | 10.12 | 0.01 |  |
| **Never married vs. married** | 7,511,483 | 10 |  |  |  |  |  |  |  |  |
| Age (years) |  |  |  |  |  |  |  |  |  | 0.20 |
| <60 | 767,579 | 5 | 0.89 | 0.81 | 0.97 | 0.01 | 22.40% | 5.16 | 0.3 |  |
| ≥60 | 93,931 | 1 | 0.54 | 0.31 | 0.95 | 0.03 | NA | 0 | NA |  |
| Others | 6,649,973 | 4 | 0.99 | 0.97 | 1.02 | 0.59 | 0.00% | 0.95 | 0.8 |  |
| Location |  |  |  |  |  |  |  |  |  | 0.51 |
| Asia | 232,631 | 3 | 0.87 | 0.66 | 1.15 | 0.31 | 0.00% | 1.62 | 0.44 |  |
| Europe | 6,658,279 | 5 | 0.85 | 0.71 | 1.01 | 0.06 | 71.40% | 14.01 | 0.01 |  |
| America | 620,573 | 2 | 0.94 | 0.86 | 1.03 | 0.18 | 0.00% | 0.62 | 0.43 |  |
| Others | NA |  |  |  |  |  |  |  |  |  |
| Follow-up years |  |  |  |  |  |  |  |  |  | 0.47 |
| <10 | 447,064 | 3 | 0.93 | 0.83 | 1.04 | 0.07 | 40.20% | 3.34 | 0.19 |  |
| ≥10 | 7,052,854 | 6 | 0.84 | 0.70 | 1.02 | 0.21 | 50.20% | 10.04 | 0.07 |  |
| Others | 11,565 | 1 | 1.44 | 0.59 | 3.50 | 0.42 | NA | 0 | NA |  |
| Publication years |  |  |  |  |  |  |  |  |  | 0.21 |
| ≤2000 | 414,446 | 3 | 0.86 | 0.69 | 1.08 | 0.19 | 58.20% | 4.78 | 0.09 |  |
| 2001-2009 | 130,920 | 4 | 0.77 | 0.61 | 0.98 | 0.03 | 0.00% | 2.55 | 0.59 |  |
| ≥2010 | 6,966,117 | 3 | 0.99 | 0.97 | 1.01 | 0.38 | 0.00% | 1.95 | 0.38 |  |

Abbreviations: N: number of studies; NA: not available.

**
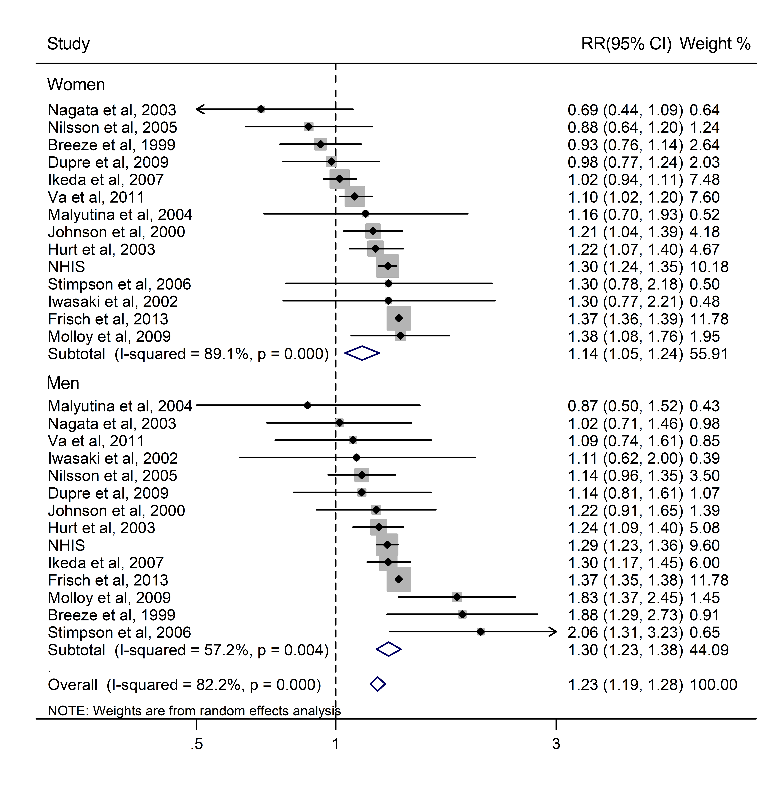

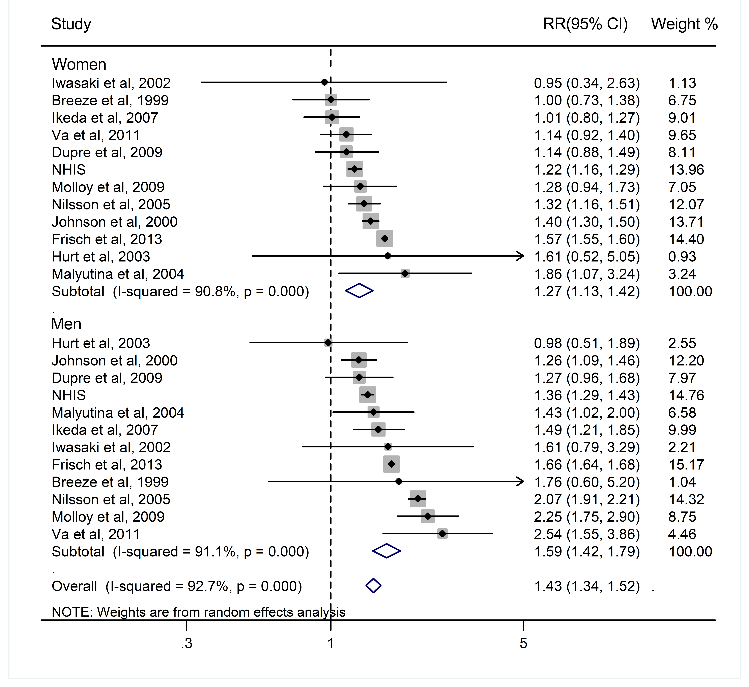

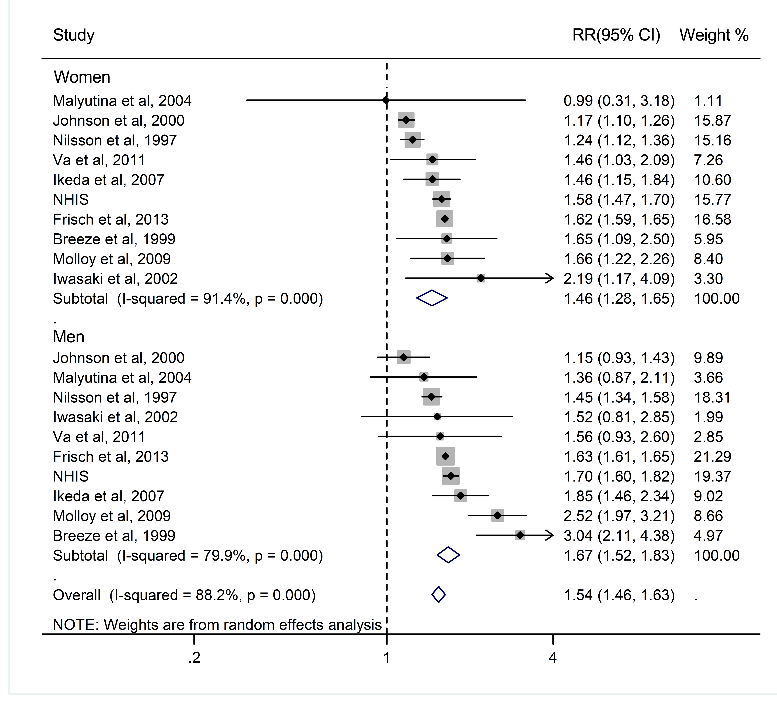
 (a) (b) (c)**

**Supplemental Figure 1. Sex-specific relative risks (RRs) for all-cause mortality, comparing widowed, divorced/separated and never married to married people: (a) Sex-specific RRs for all-cause mortality, comparing widowed to married people; (b) Sex-specific RRs for all-cause mortality, comparing divorced/separated to married people; (c) Sex-specific RRs for all-cause mortality, comparing never married to married people.**

**
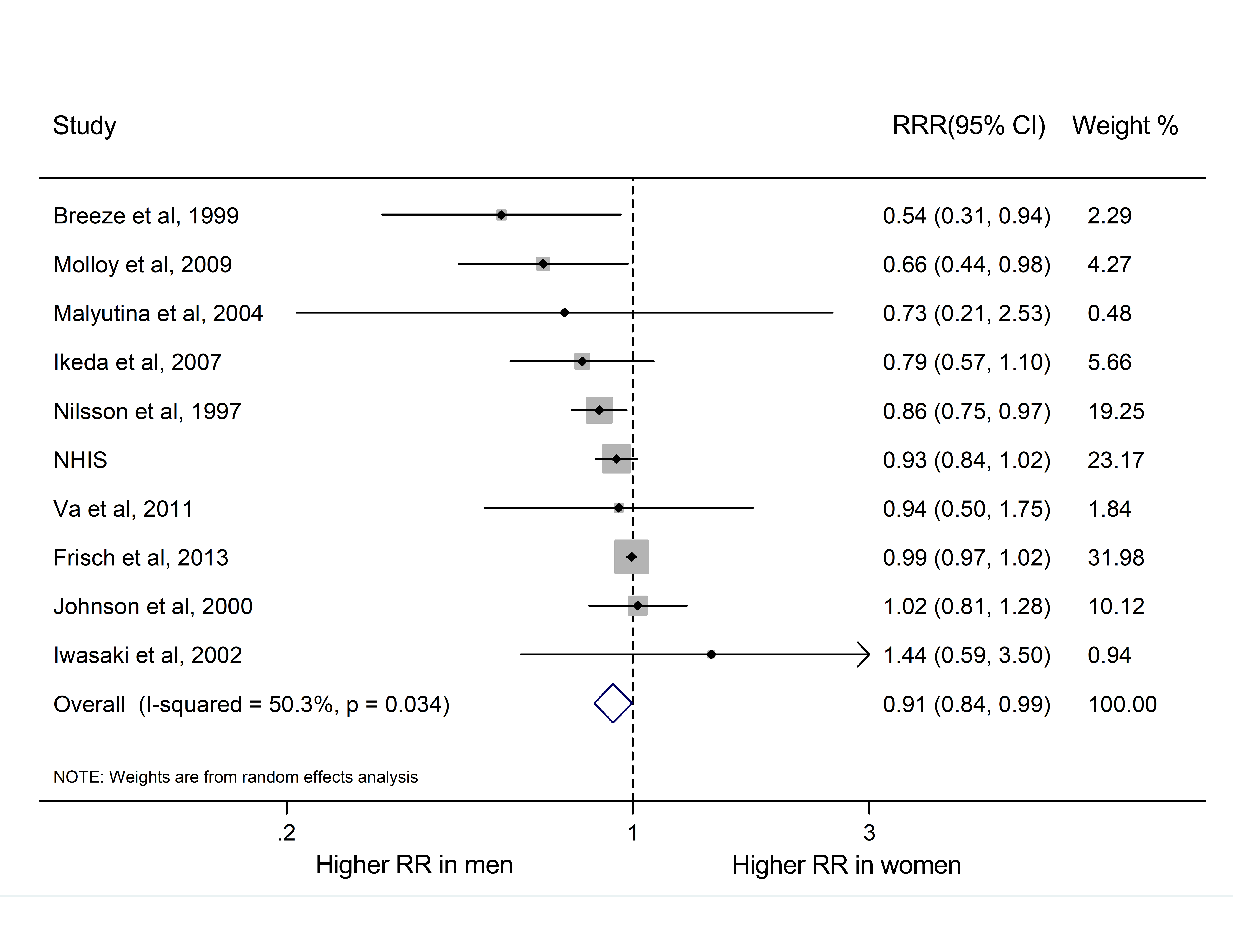

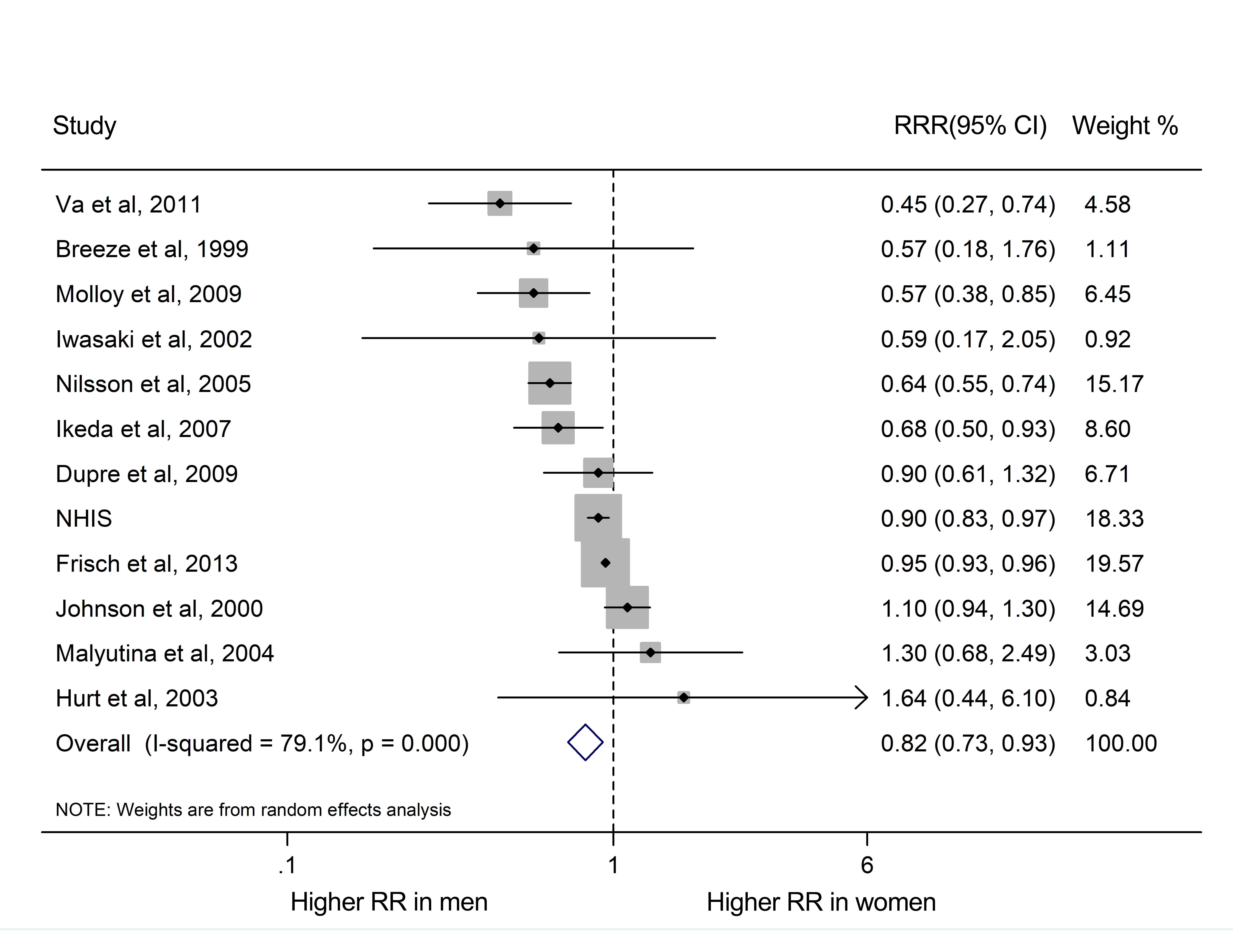

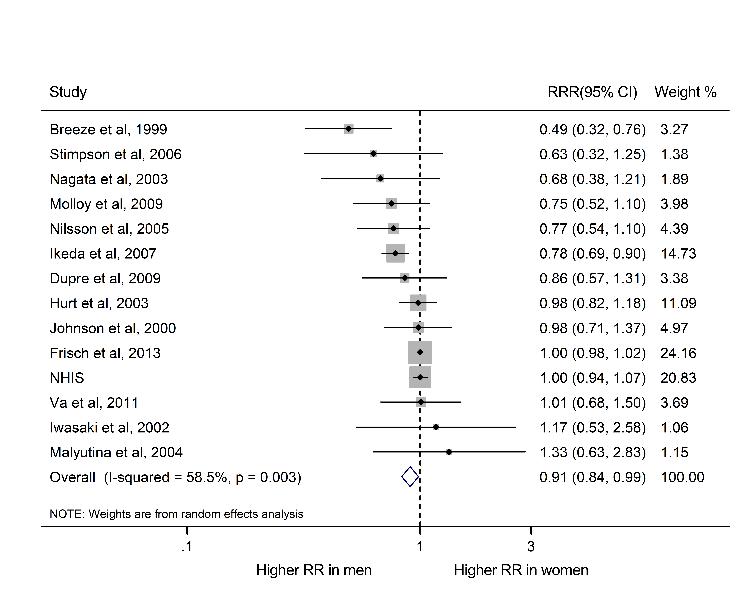
 (a) (b) (c)**

**Supplemental Figure 2. Women-to-men ratios of relative risks (RRRs) for all-cause mortality comparing widowed, divorced/separated and never married to married people: (a) Women-to-men RRRs for all-cause mortality comparing widowed to married people; (b) Women-to-men RRRs for all-cause mortality comparing divorced/separated to married people; (c) Women-to-men RRRs for all-cause mortality comparing never married to married people.**

**(a) (b)**

**
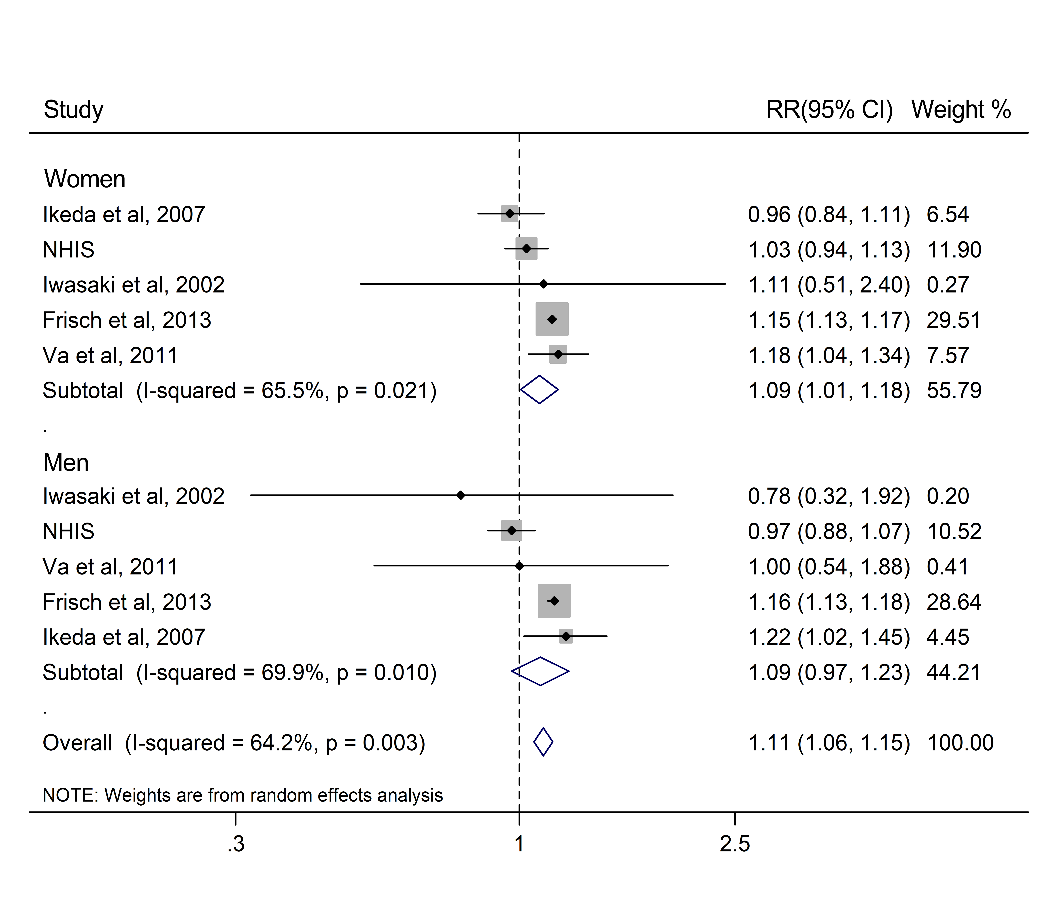
**
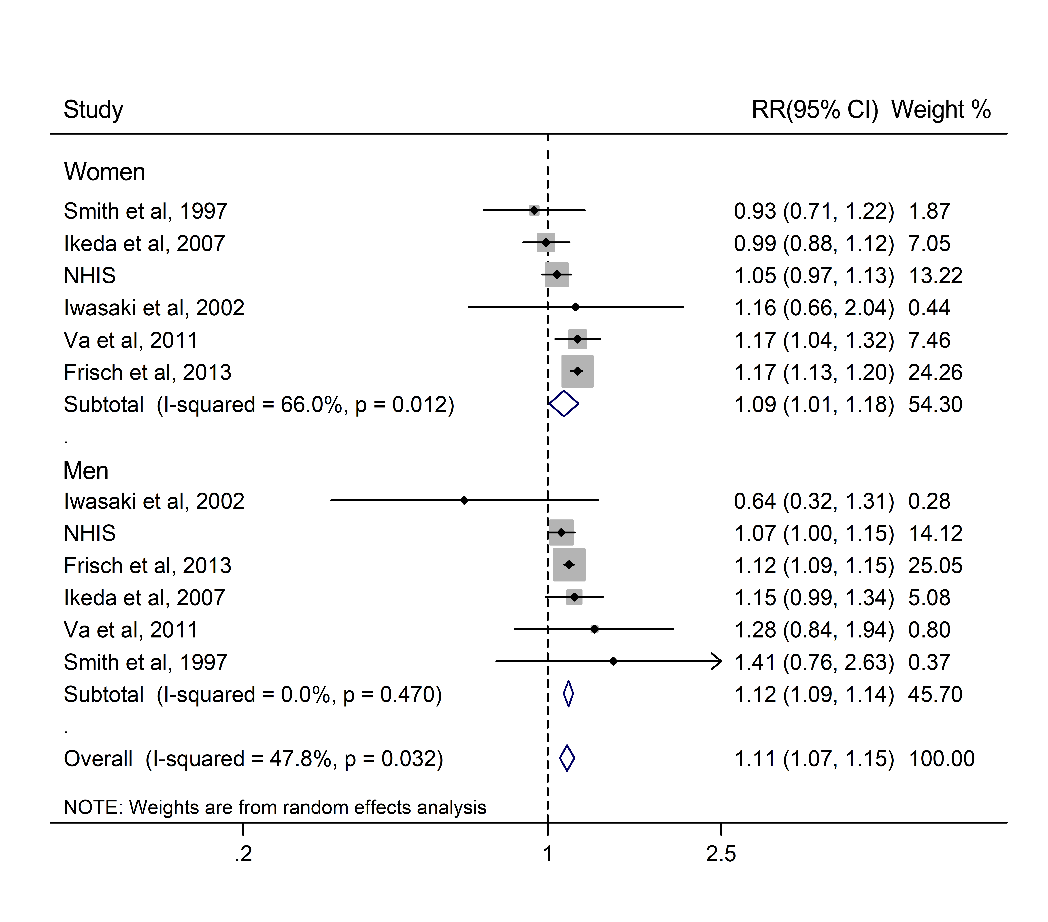


**(c) (d)**

**
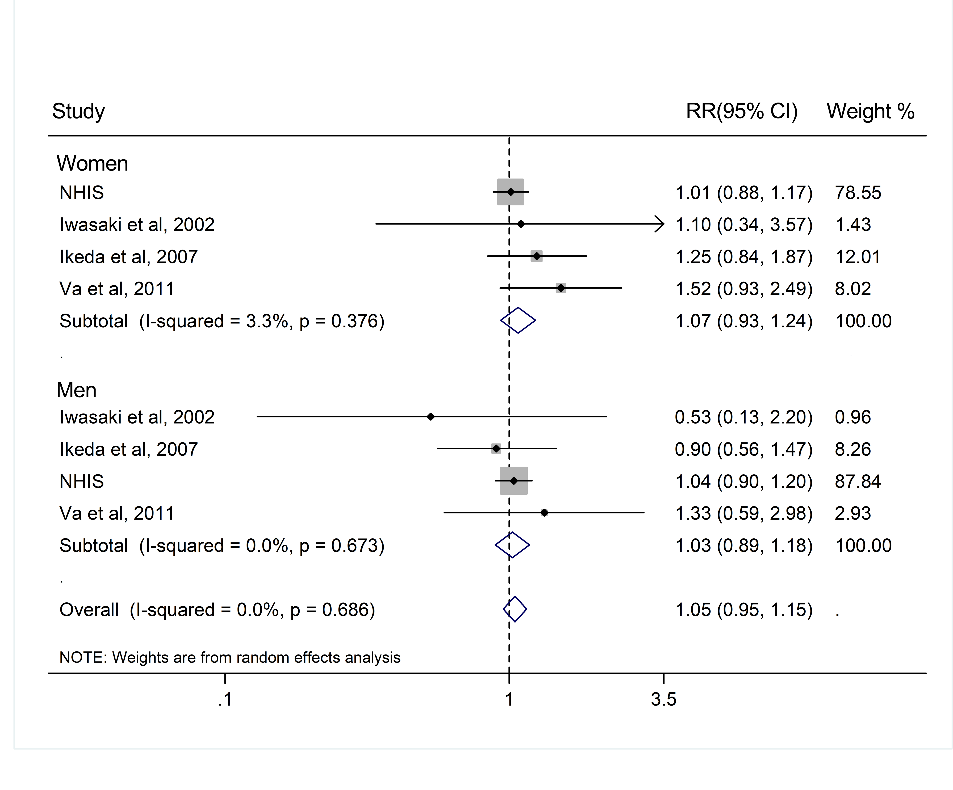

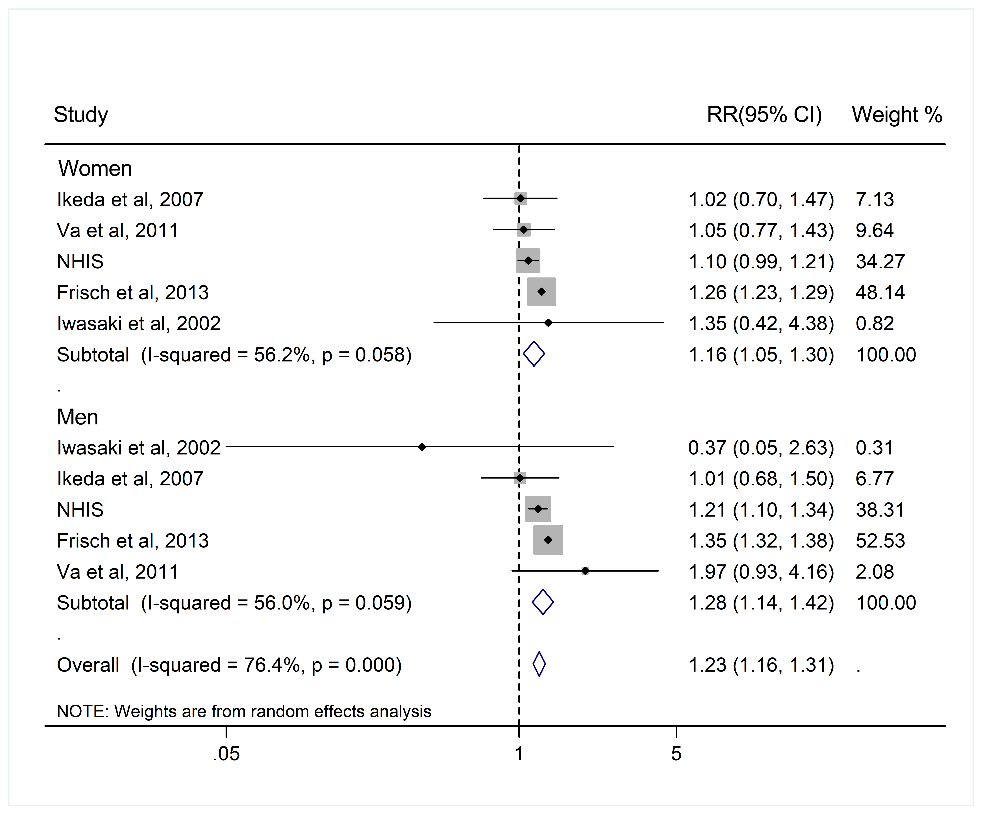
**

**Supplemental Figure 3. Sex-specific relative risks (RRs) for cancer mortality, comparing non-married, widowed, divorced/separated and never married to married people: (a) Sex-specific RRs for cancer mortality, comparing non-married to married people; (b) Sex-specific RRs for cancer mortality, comparing widowed to married people; (c) Sex-specific RRs for cancer mortality, comparing divorced/separated to married people; (d) Sex-specific RRs for cancer mortality, comparing never married to married people.**

**(a) (b)**

**
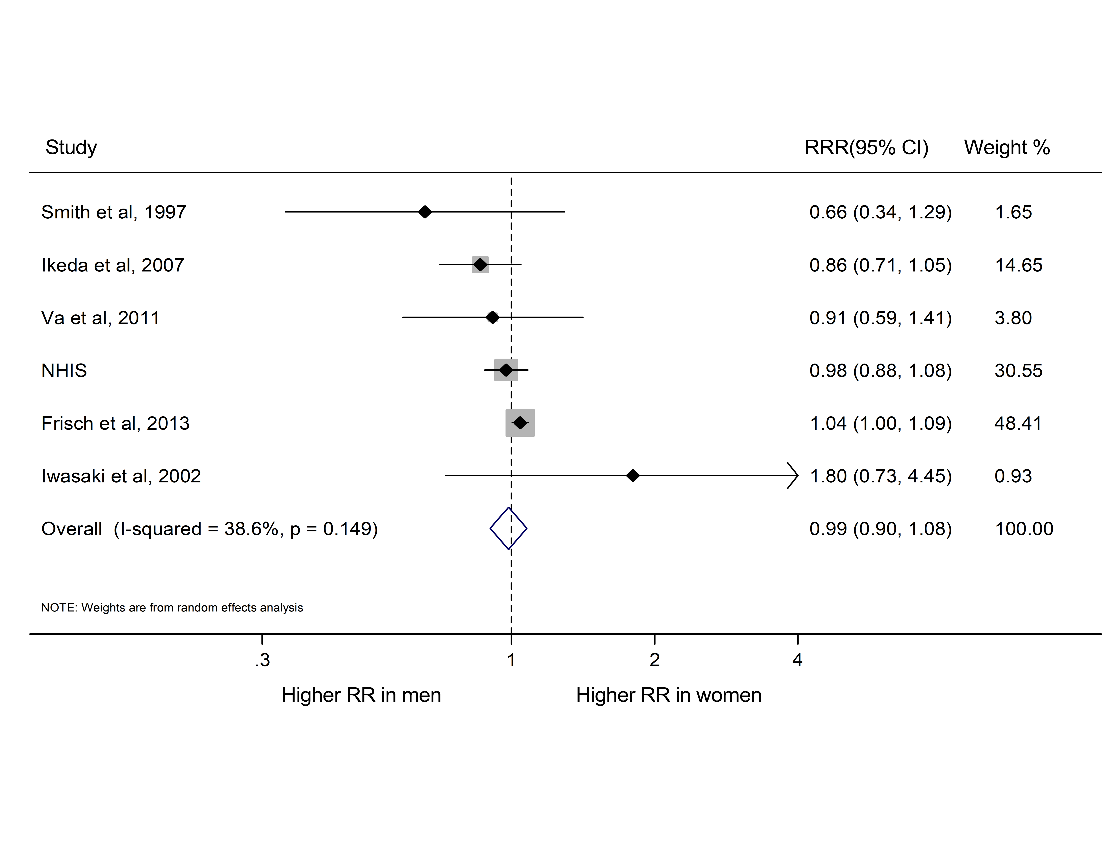
**
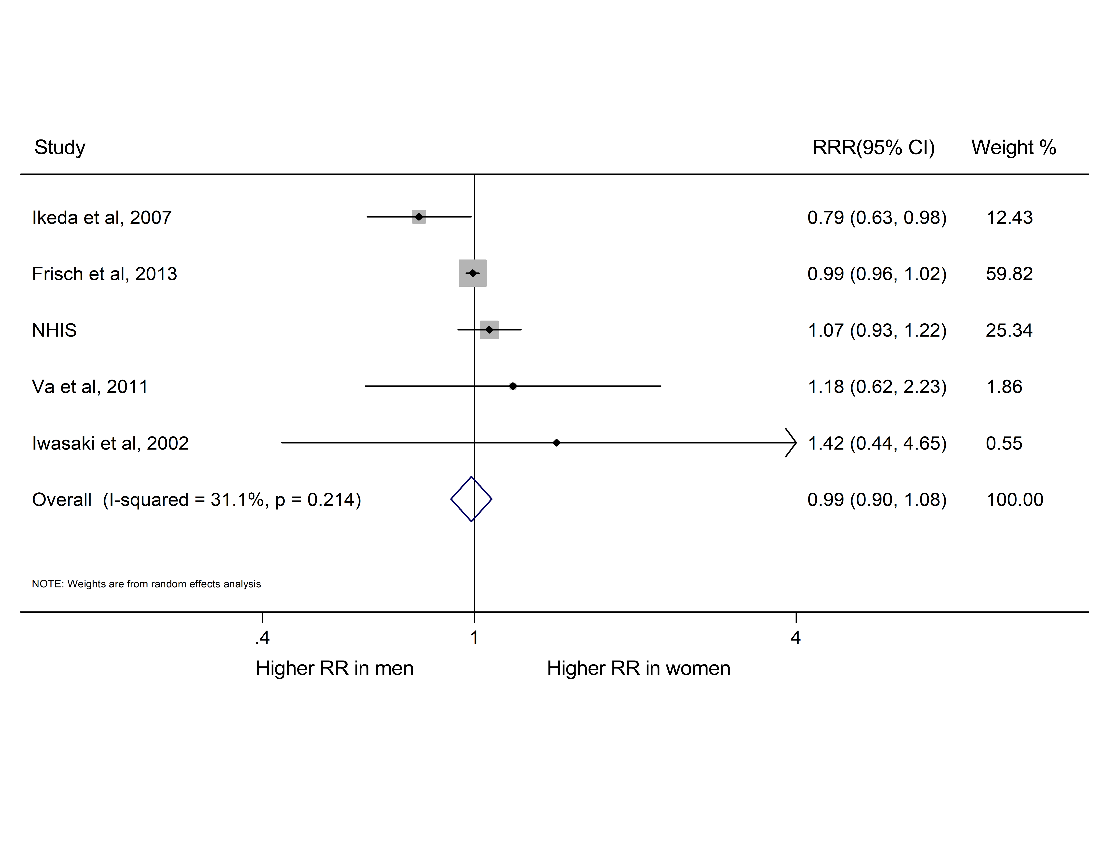


**
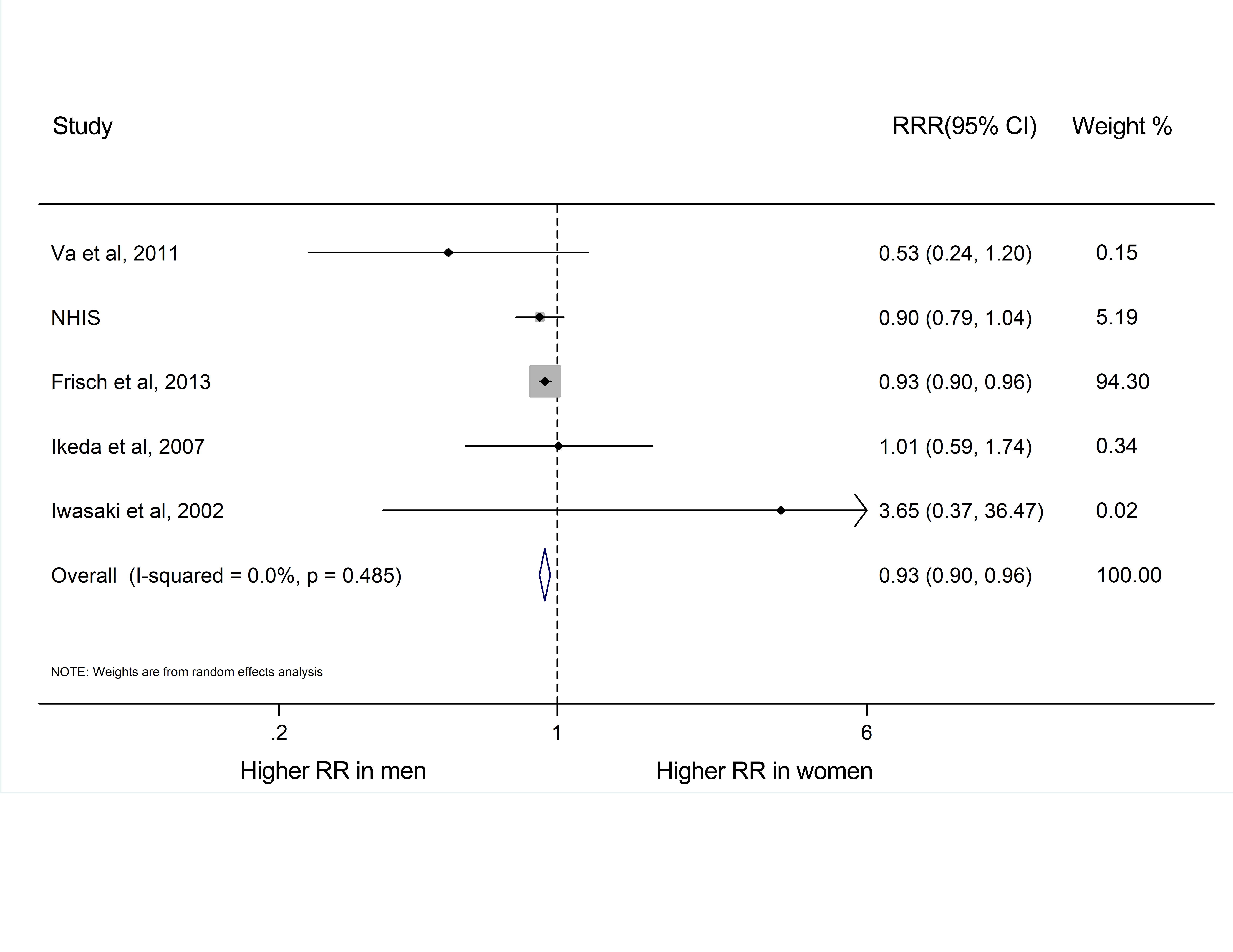

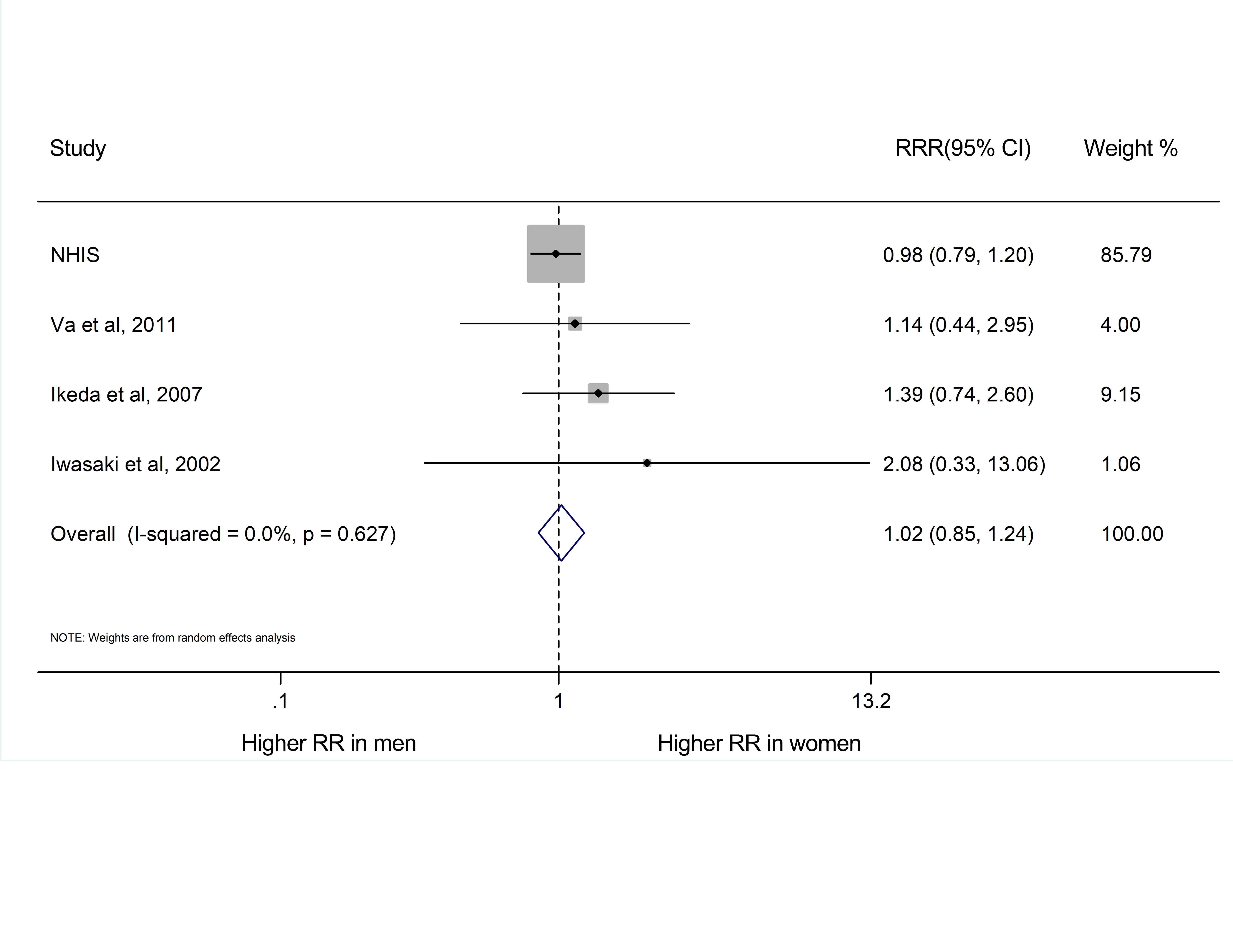
 (c) (d)**

**Supplemental Figure 4. Women-to-men ratios of relative risks (RRRs) for cancer mortality comparing non-married, widowed, divorced/separated and never married to married people: (a) Women-to-men RRRs for cancer mortality comparing non-married to married people; (b) Women-to-men RRRs for cancer mortality comparing widowed to married people; (c) Women-to-men RRRs for cancer mortality comparing divorced/separated to married people; (d) Women-to-men RRRs for cancer mortality comparing never married to married people.**

**
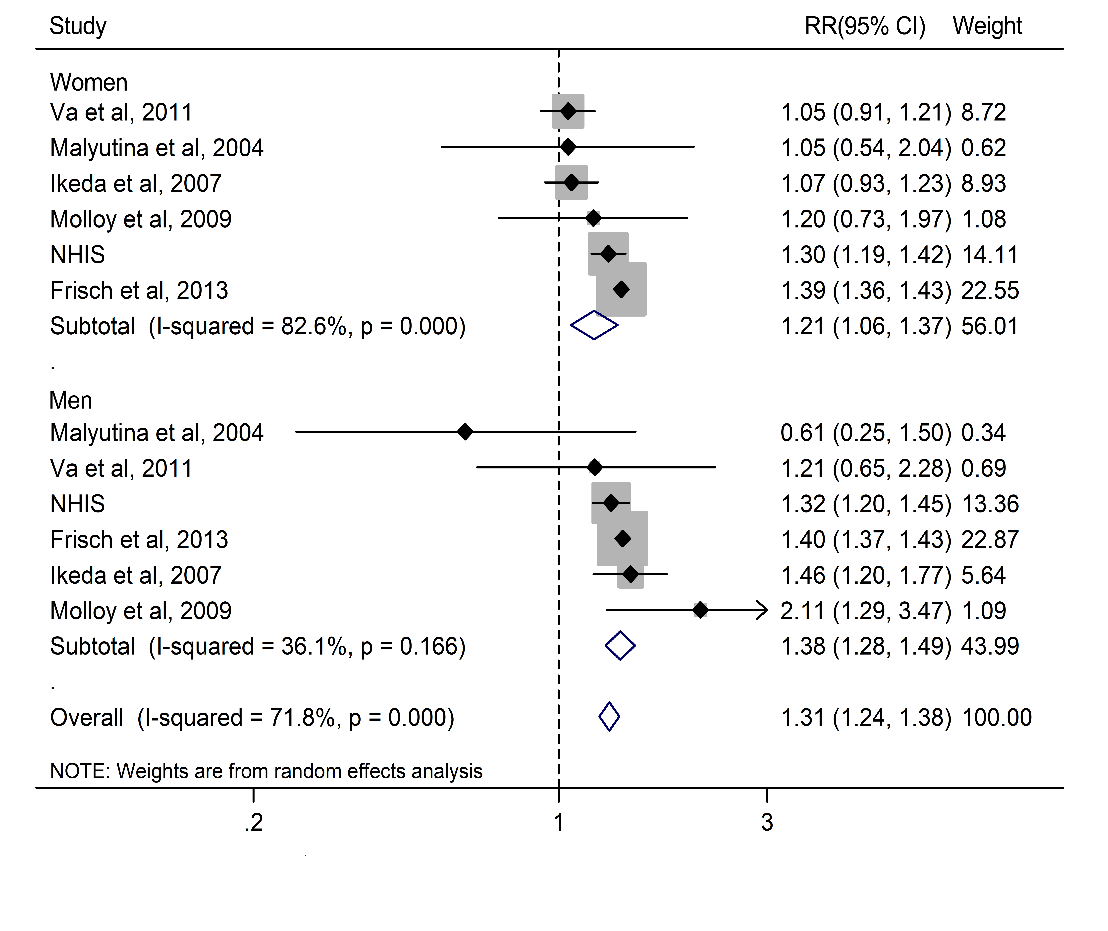
**
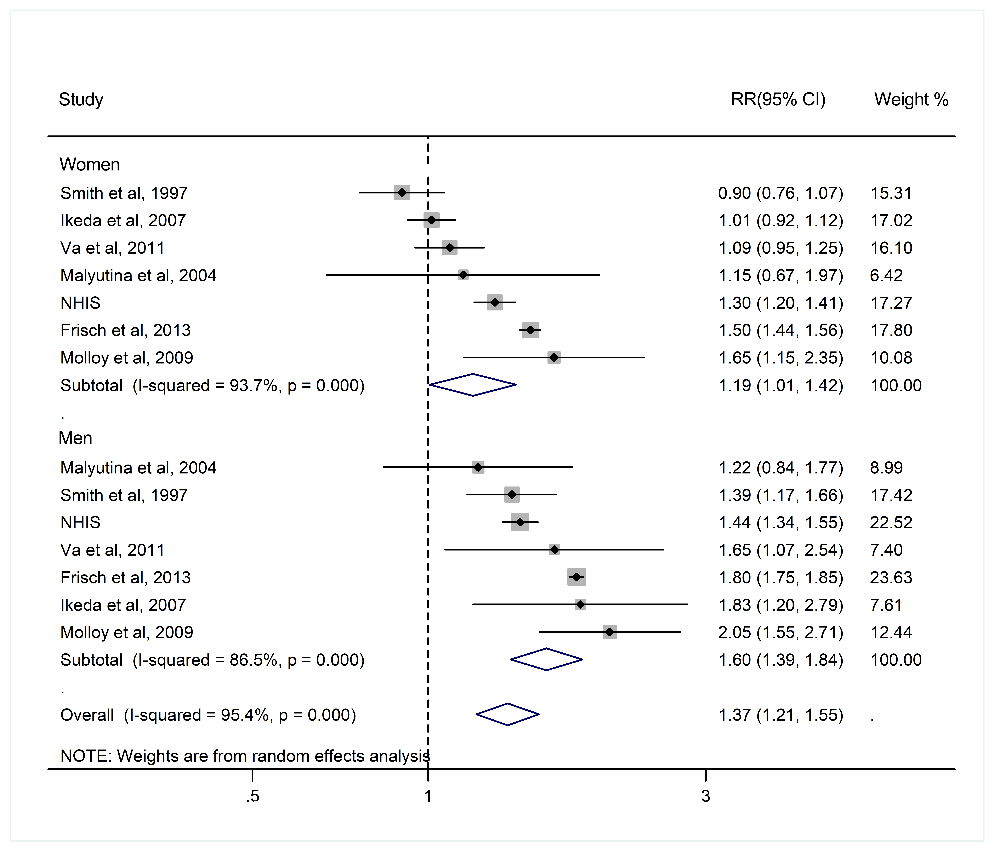
 **(a) (b)**

**
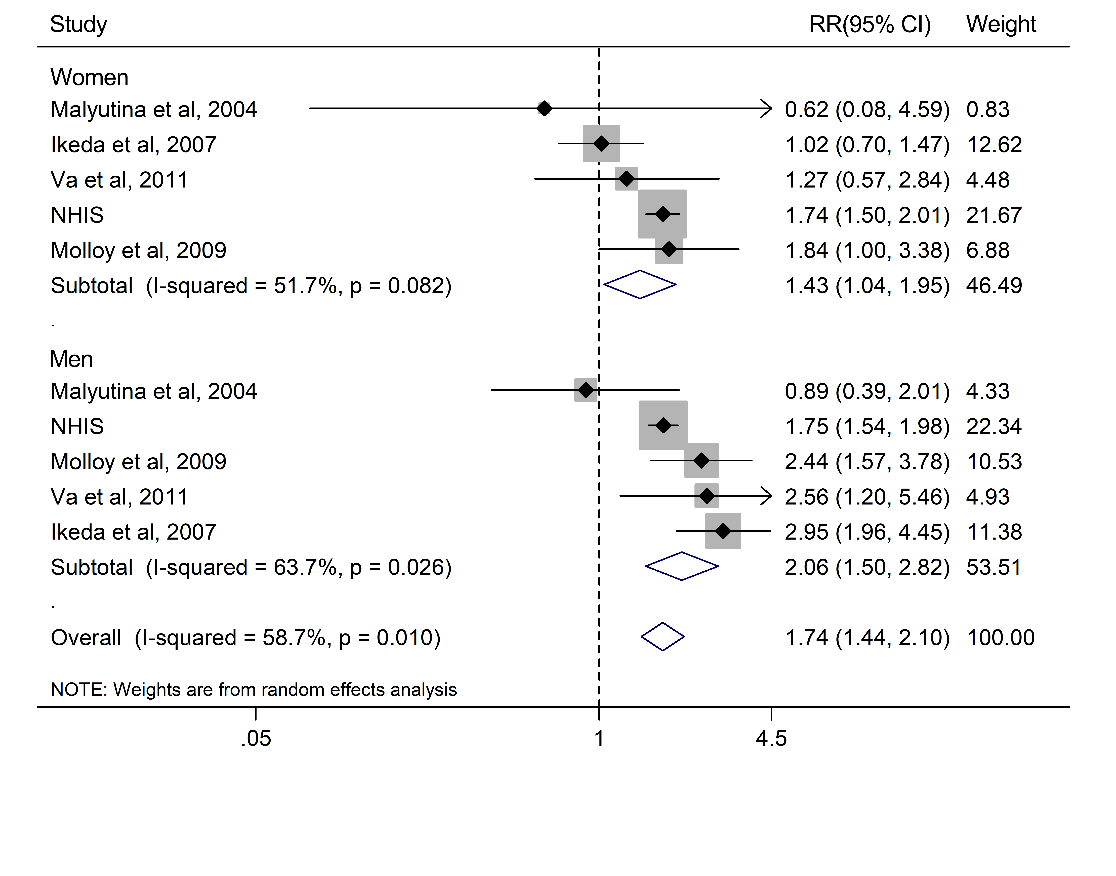

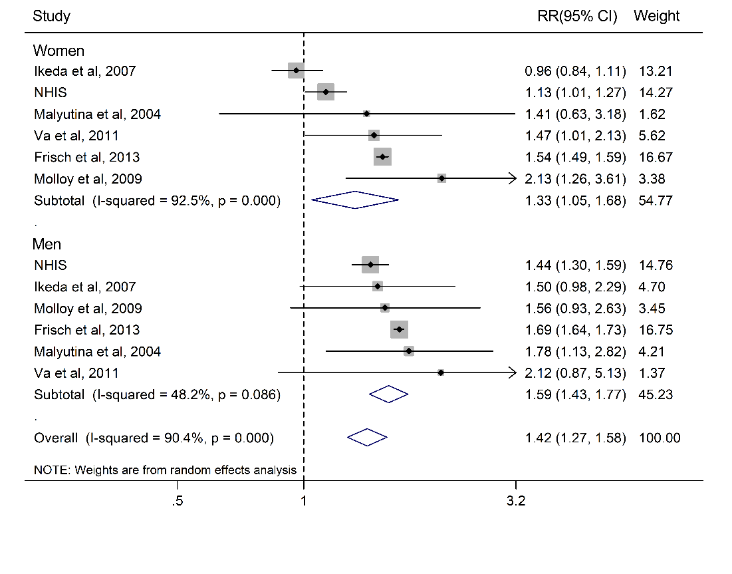
(c) (d)**

**Supplemental Figure 5. Sex-specific relative risks (RRs) for cardiovascular (CV) mortality, comparing non-married, widowed, divorced/separated and never married to married people: (a) Sex-specific RRs for CV mortality, comparing non-married to married people; (b) Sex-specific RRs for CV mortality, comparing widowed to married people; (c) Sex-specific RRs for CV mortality, comparing divorced/separated to married people; (d) Sex-specific RRs for CV mortality, comparing never married to married people.**

**(a) (b)**

**
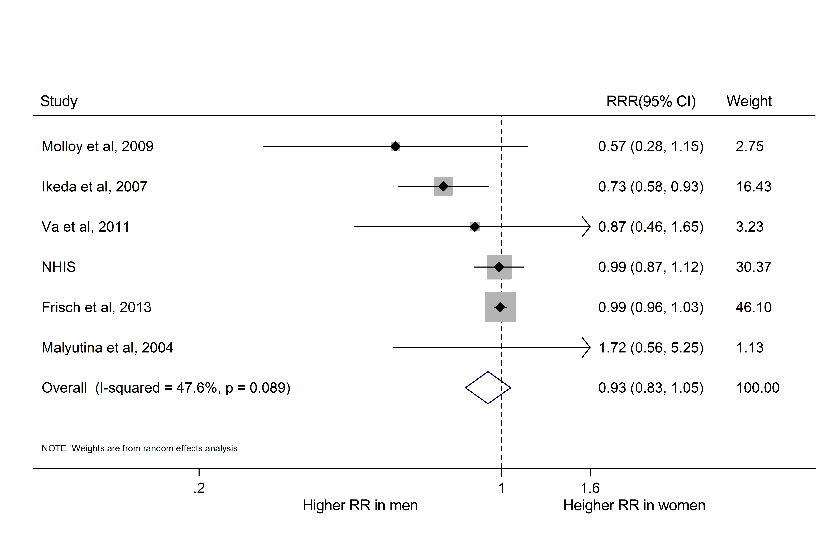

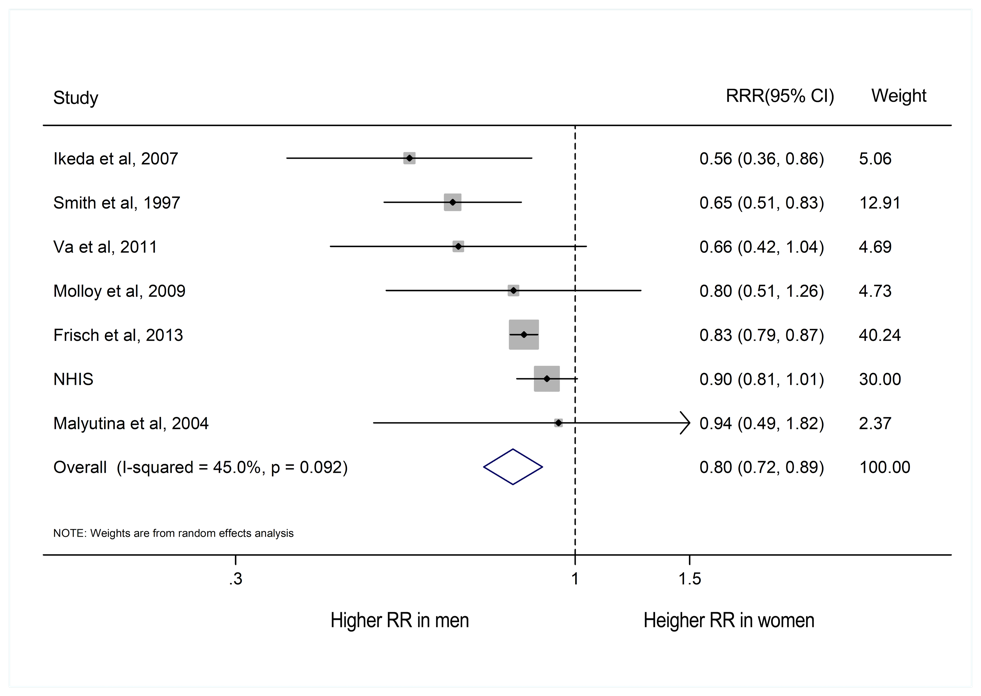
**

**
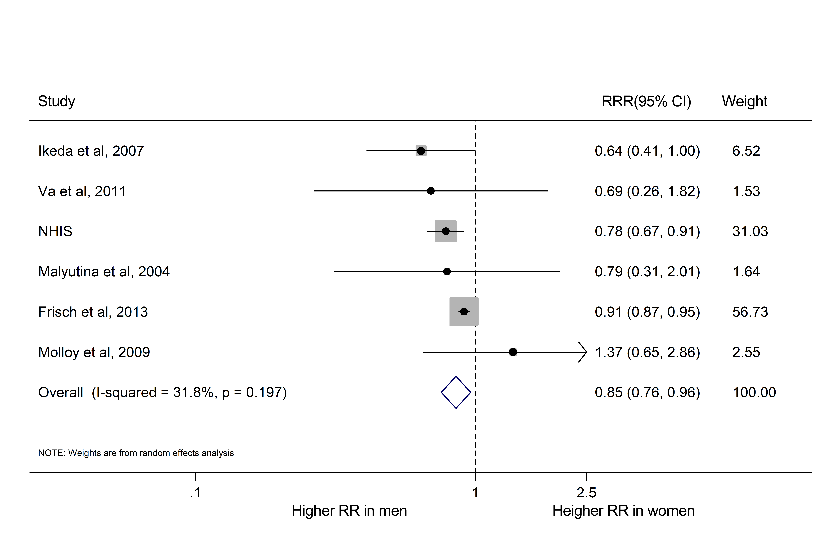
**

**(c) (d)
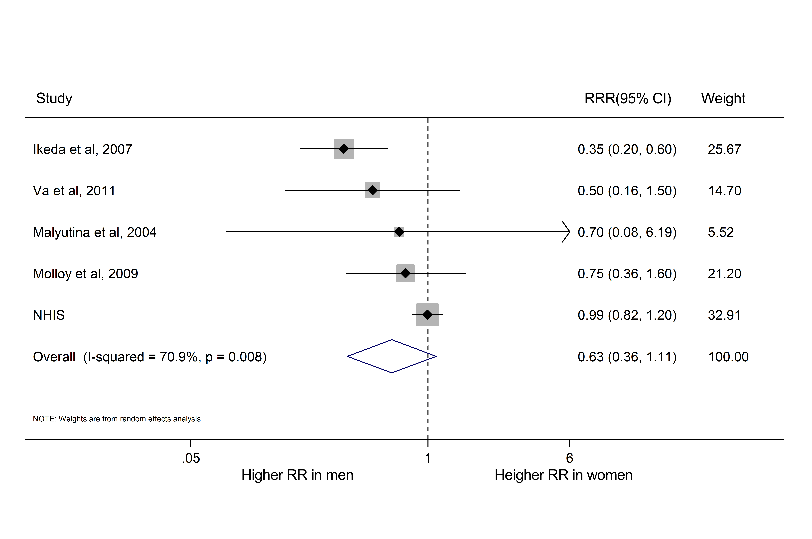
**

**Supplemental Figure 6. Women-to-men ratios of relative risks (RRRs) for cardiovascular (CV) mortality comparing non-married, widowed, divorced/separated and never married to married people: (a) Women-to-men RRRs for CV mortality comparing non-married to married people; (b) Women-to-men RRRs for CV mortality comparing widowed to married people; (c) Women-to-men RRRs for CV mortality comparing divorced/separated to married people; (d) Women-to-men RRRs for CV mortality comparing never married to married people.**


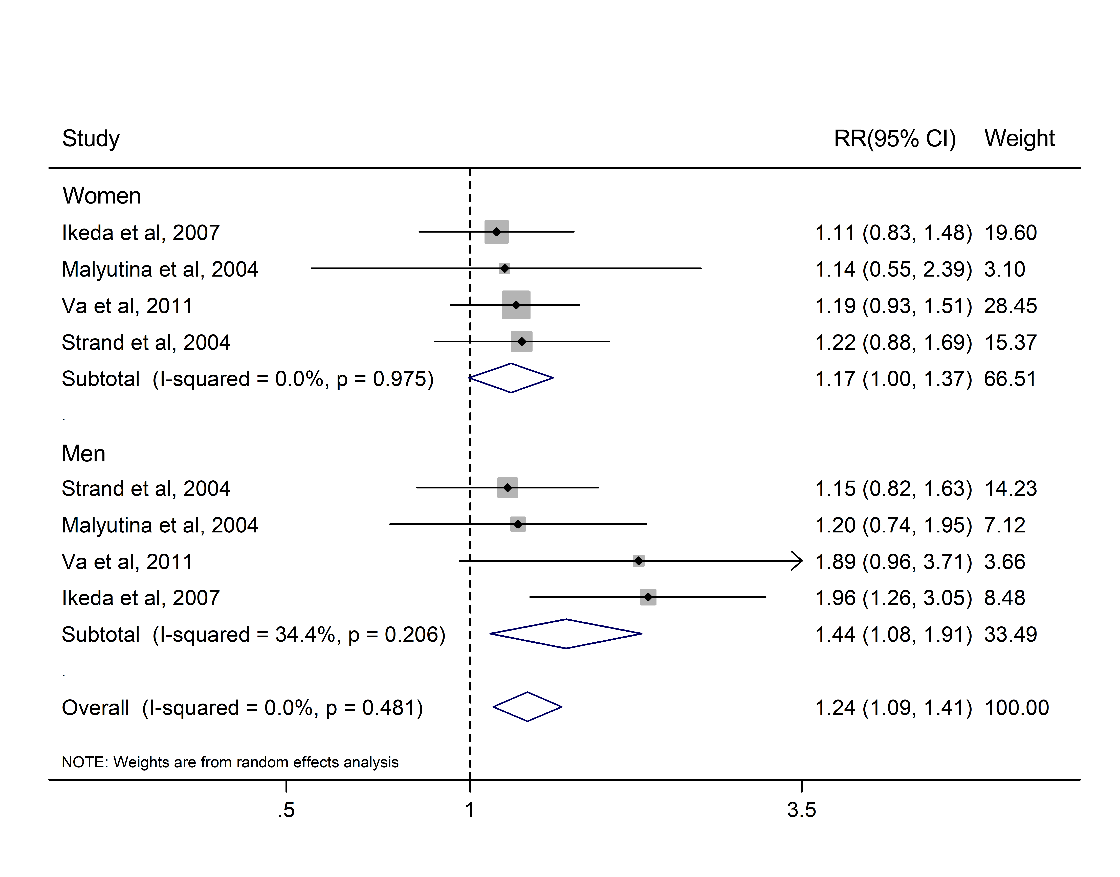
**
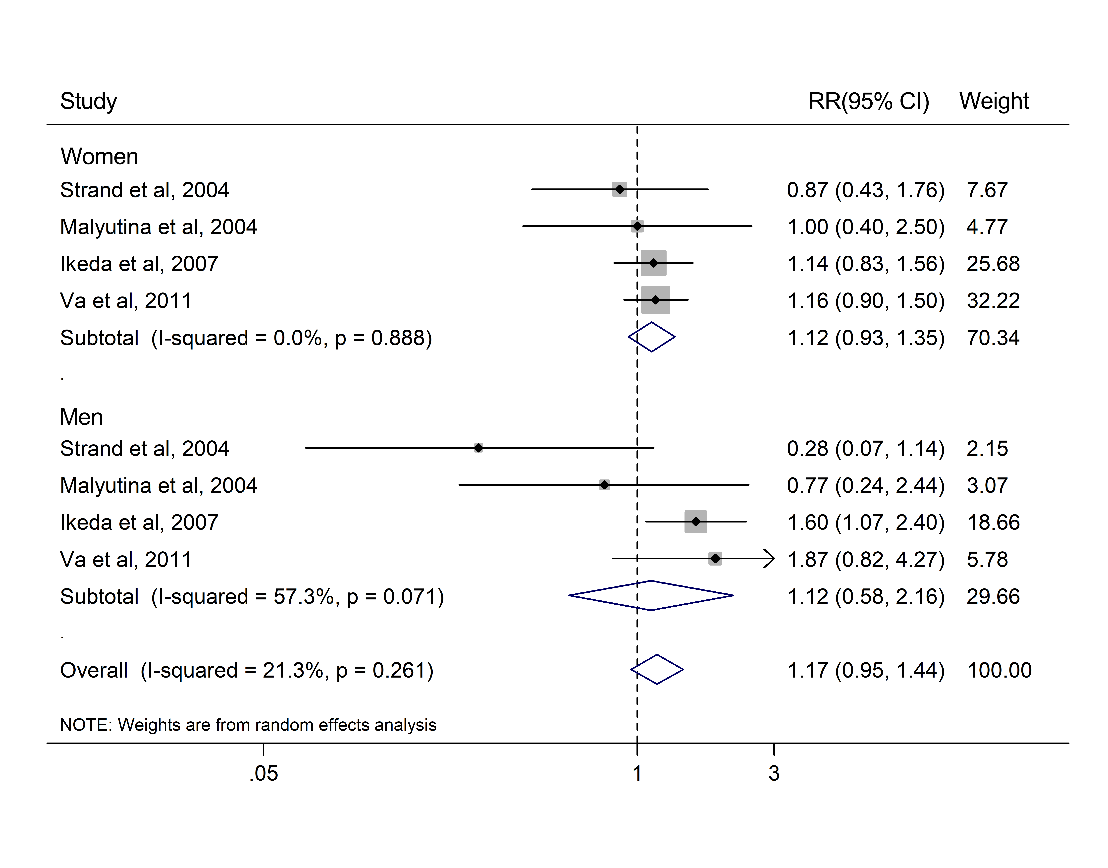
 (a) (b)**

**
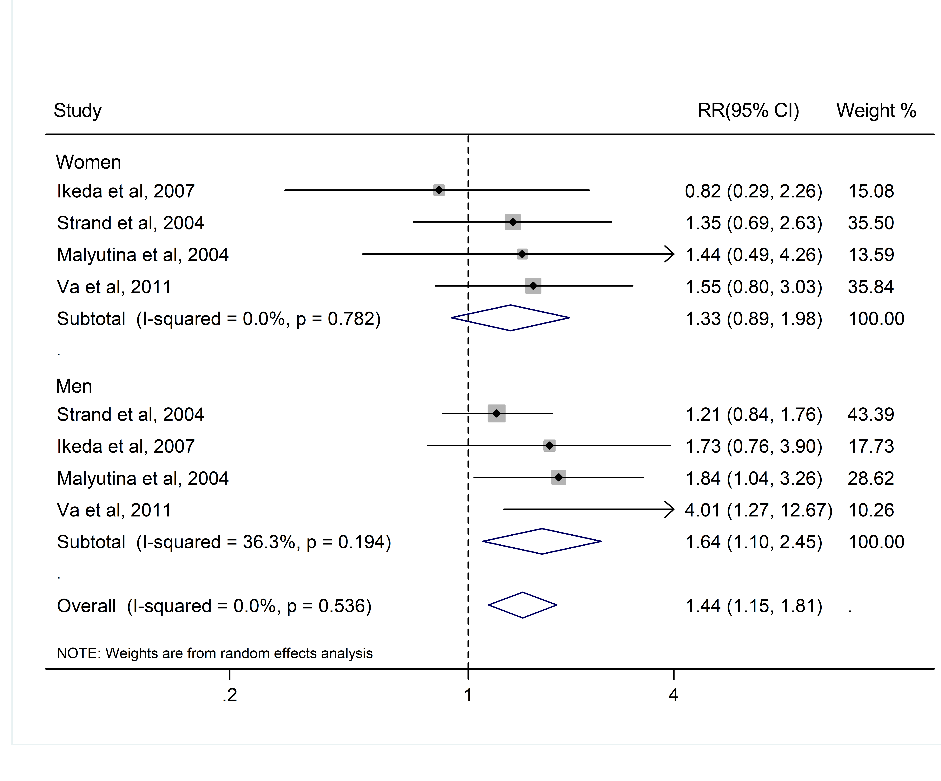

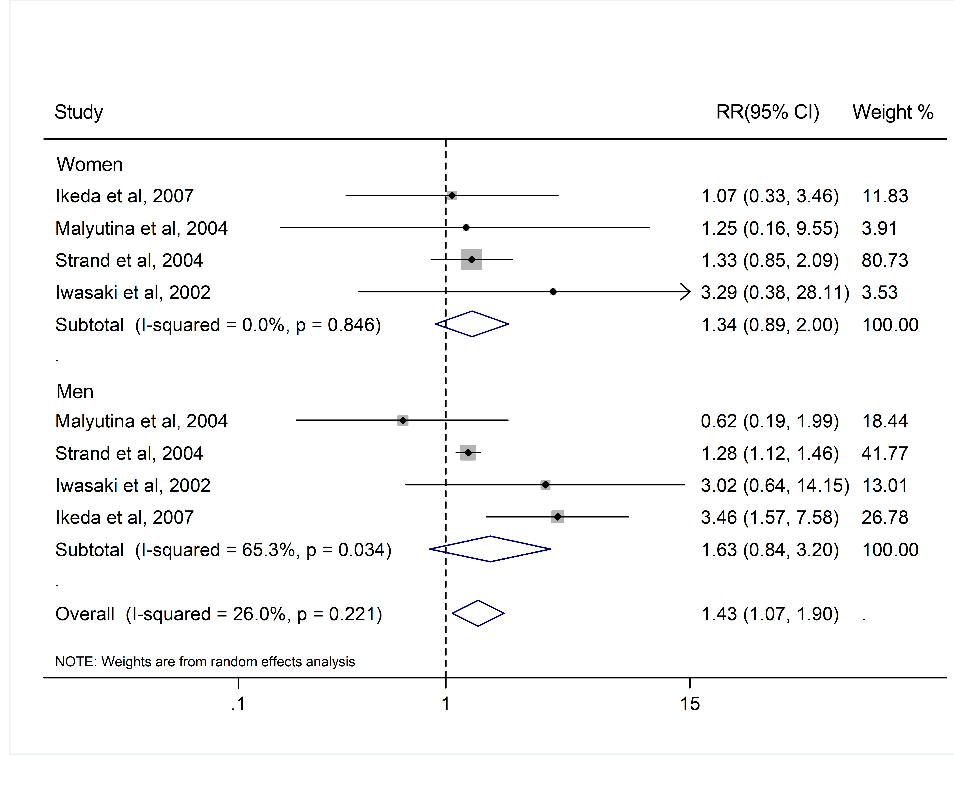
 (c) (d)**

**Supplemental Figure 7. Sex-specific relative risks (RRs) for coronary heart disease (CHD) mortality, comparing non-married, widowed, divorced/separated and never married to married people: (a) Sex-specific RRs for CHD mortality, comparing non-married to married people; (b) Sex-specific RRs for CHD mortality, comparing widowed to married people; (c) Sex-specific RRs for CHD mortality, comparing divorced/separated to married people; (d) Sex-specific RRs for CHD mortality, comparing never married to married people.**


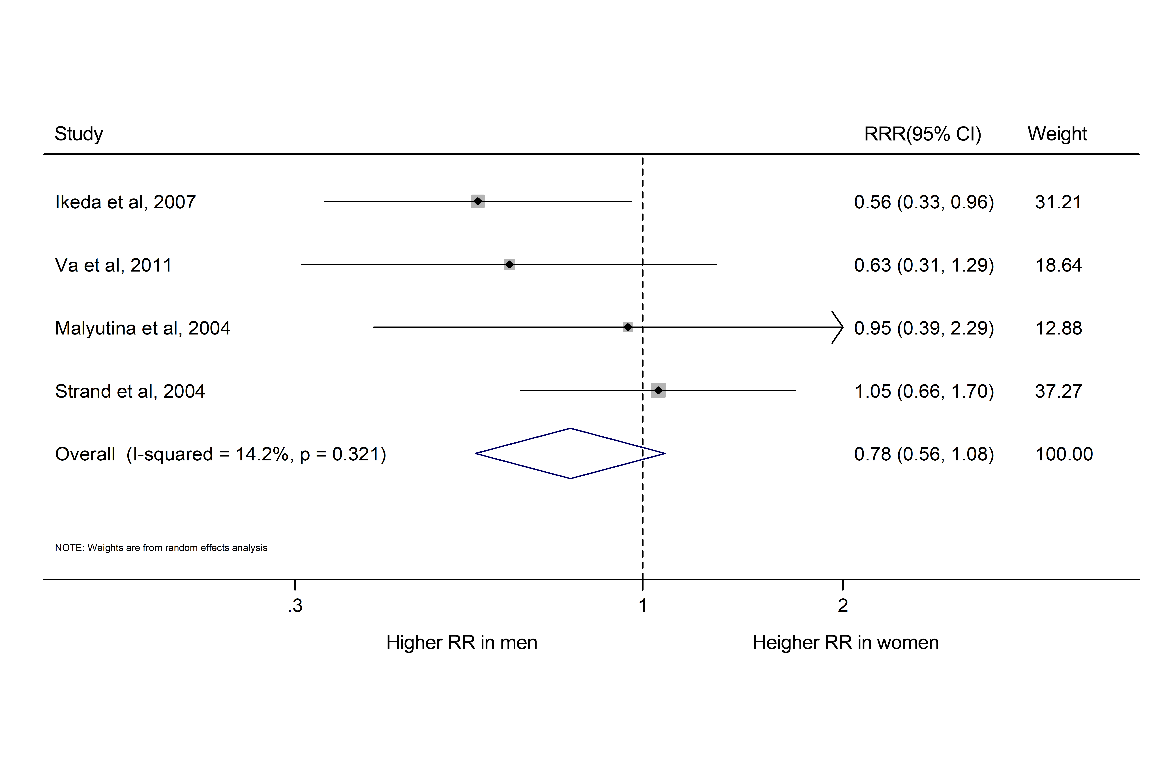


**(a) (b)
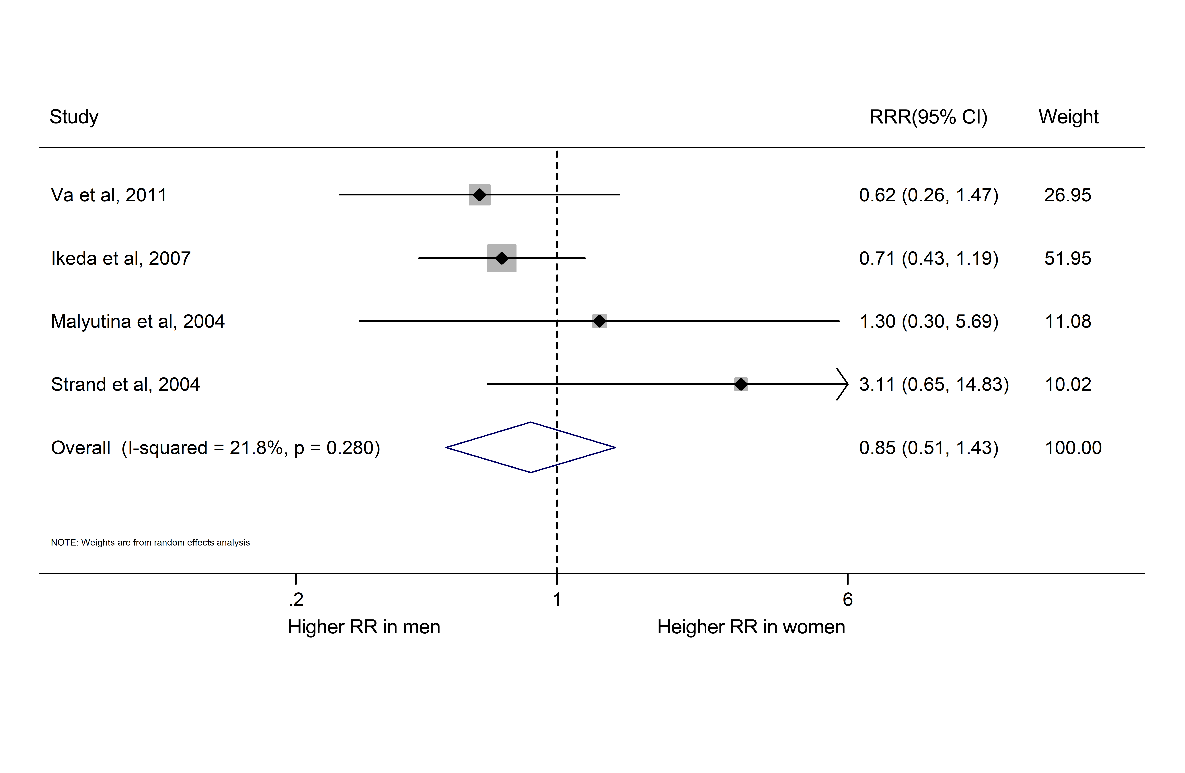
**

**(c) (d)**

**
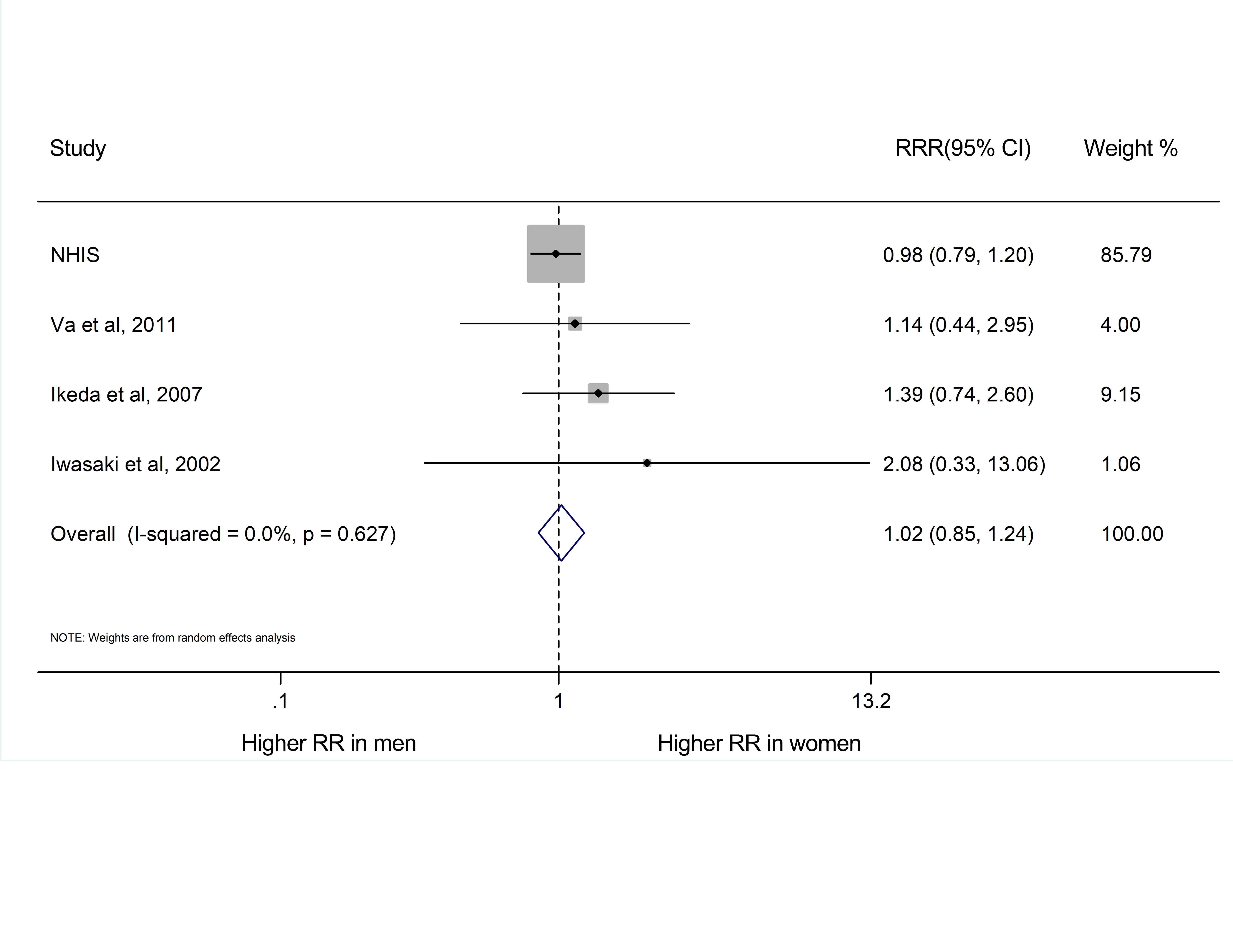

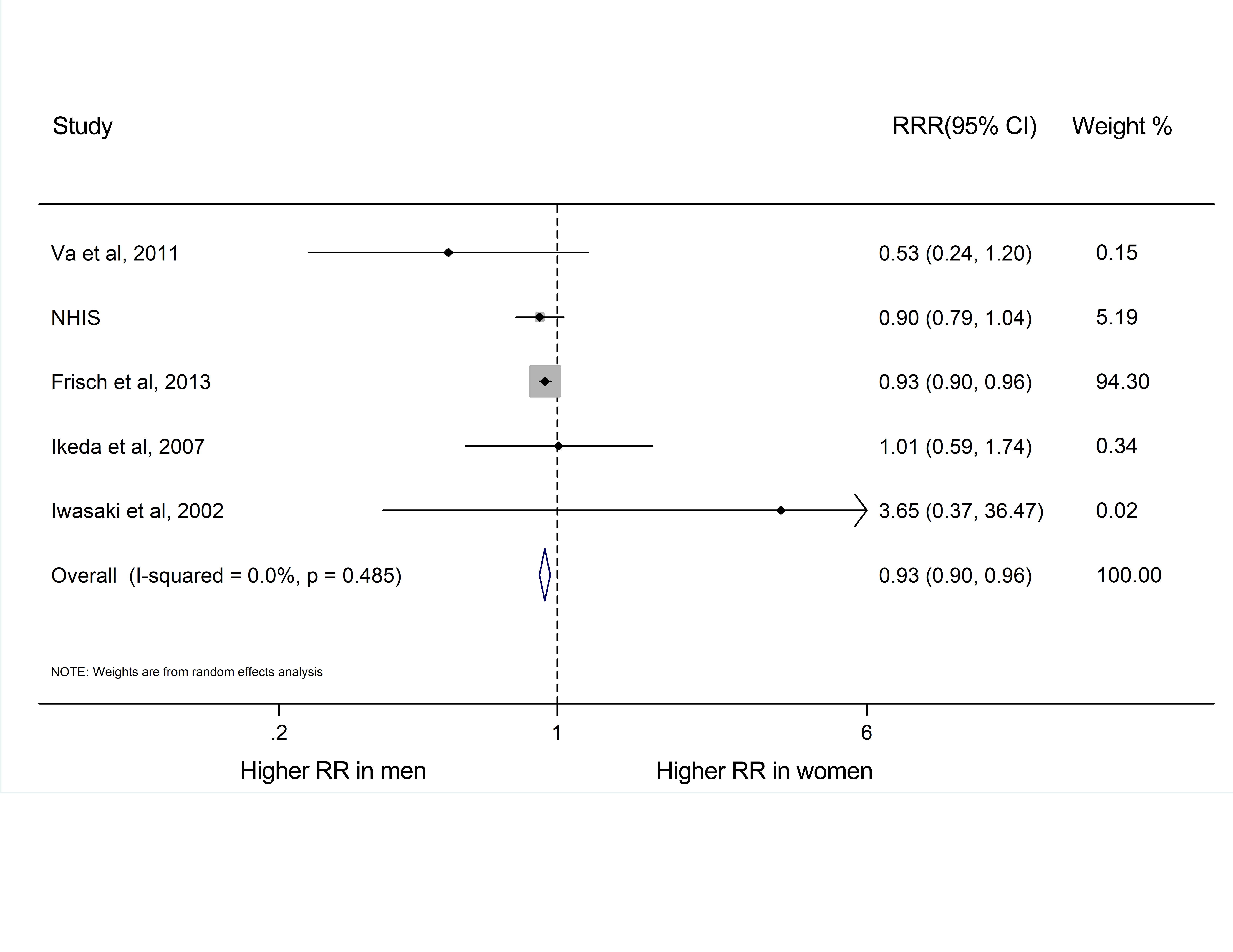
**

**Supplemental Figure 8. Women-to-men ratios of relative risks (RRRs) for coronary heart disease (CHD) mortality comparing non-married, widowed, divorced/separated and never married to married people: (a) Women-to-men RRRs for CHD mortality comparing non-married to married people; (b) Women-to-men RRRs for CHD mortality comparing widowed to married people; (c) Women-to-men RRRs for CHD mortality comparing divorced/separated to married people; (d) Women-to-men RRRs for CV mortality comparing never married to married people.**

**
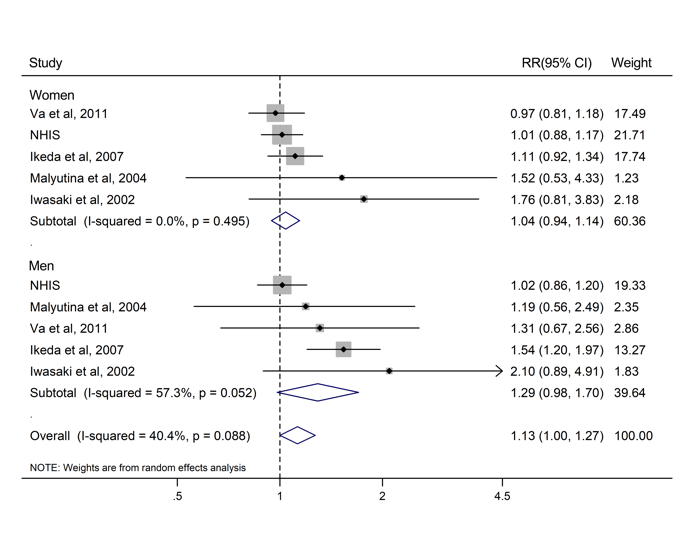

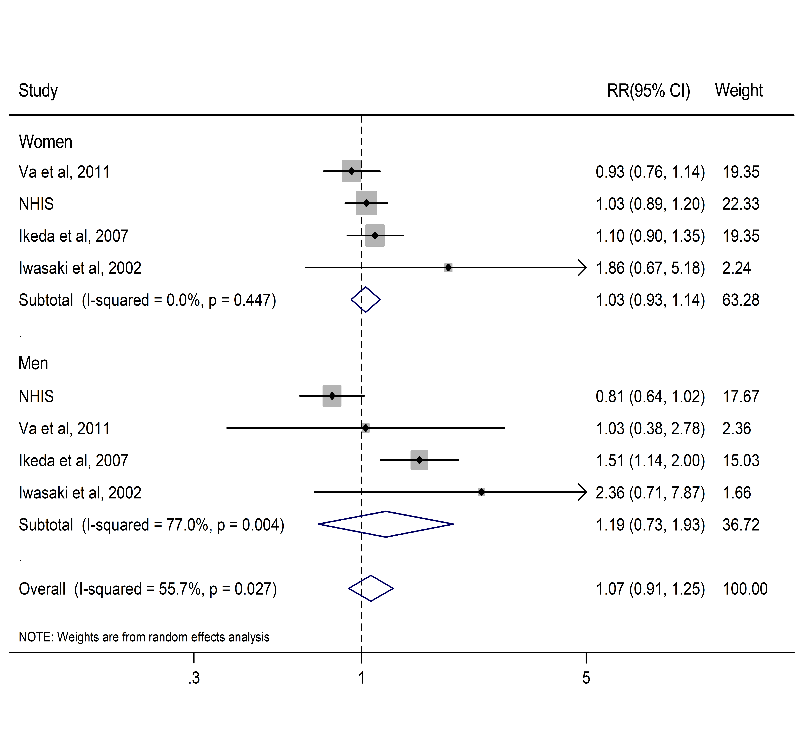
 (a) (b)**

**
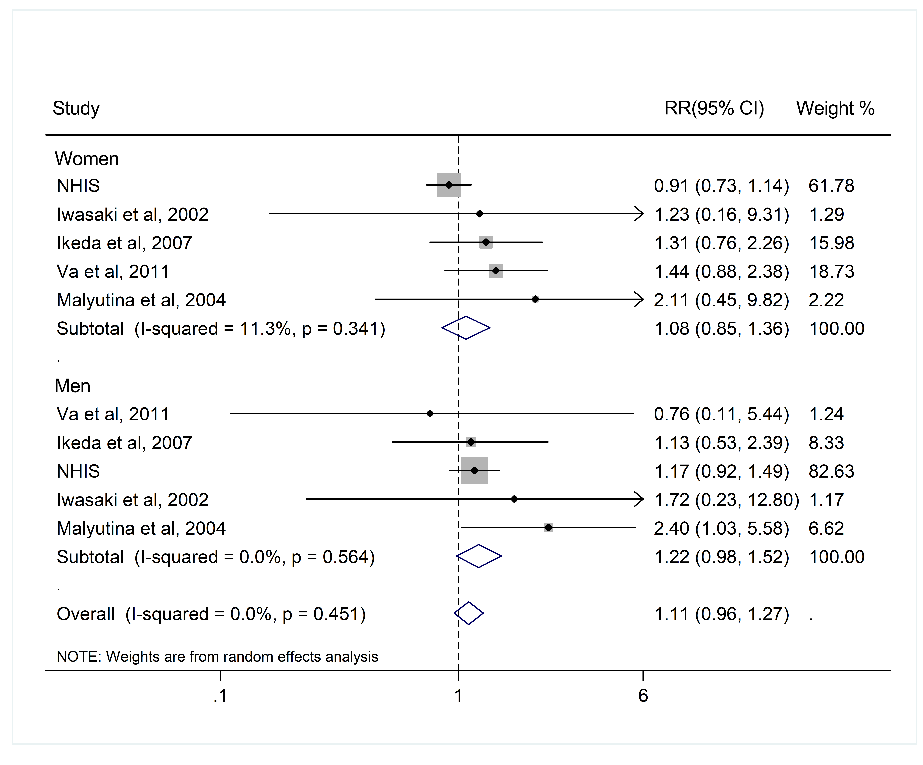

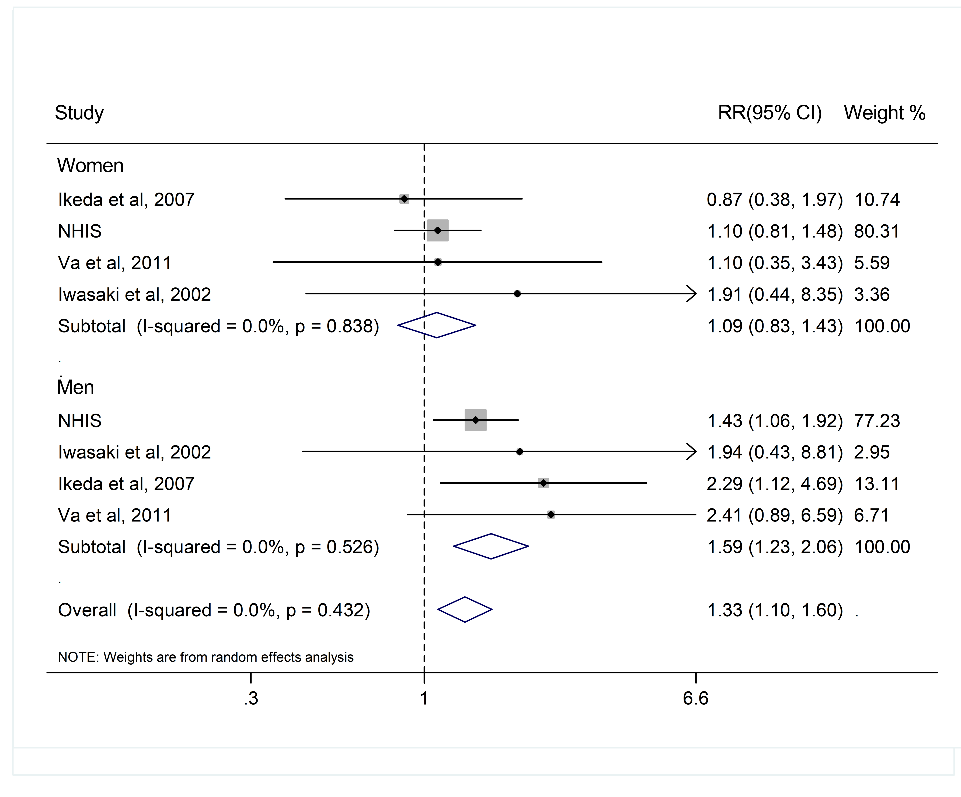
 (c) (d)**

**Supplemental Figure 9. Sex-specific relative risks (RRs) for stroke mortality, comparing non-married, widowed, divorced/separated and never married to married people: (a) Sex-specific RRs for stroke mortality, comparing non-married to married people; (b) Sex-specific RRs for stroke mortality, comparing widowed to married people; (c) Sex-specific RRs for stroke mortality, comparing divorced/separated to married people; (d) Sex-specific RRs for stroke mortality, comparing never married to married people**

**(a) (b)**


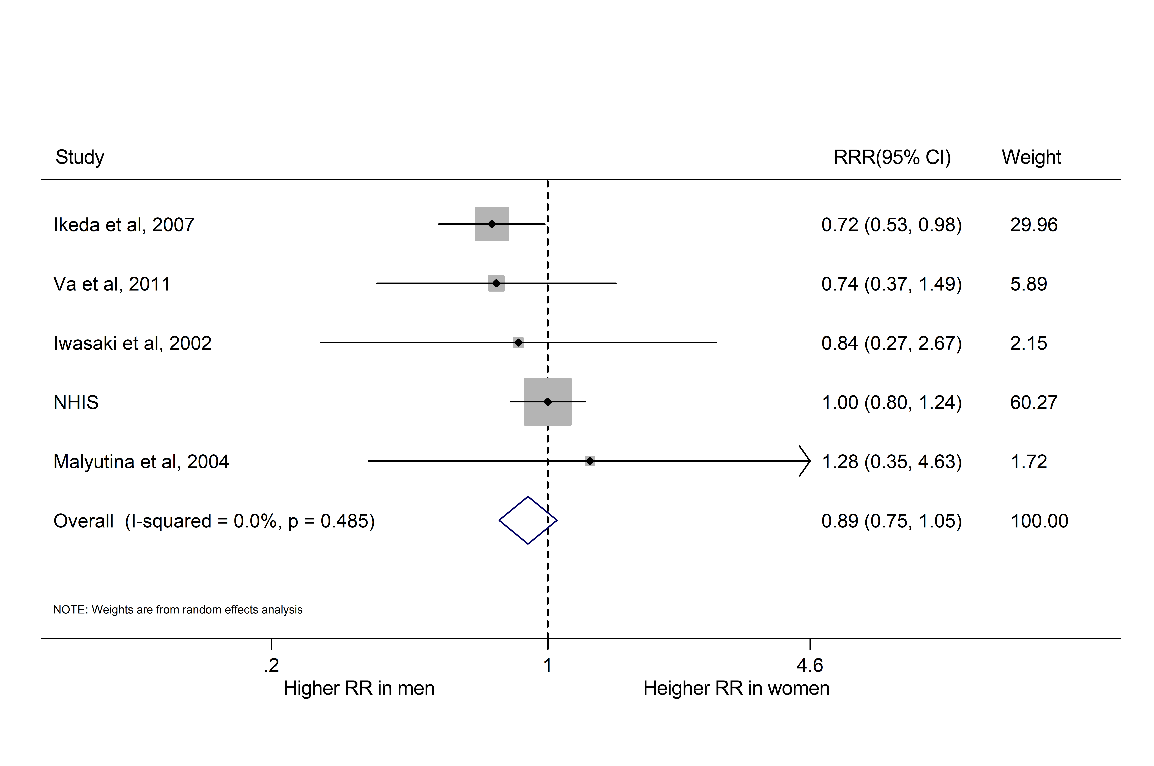
**
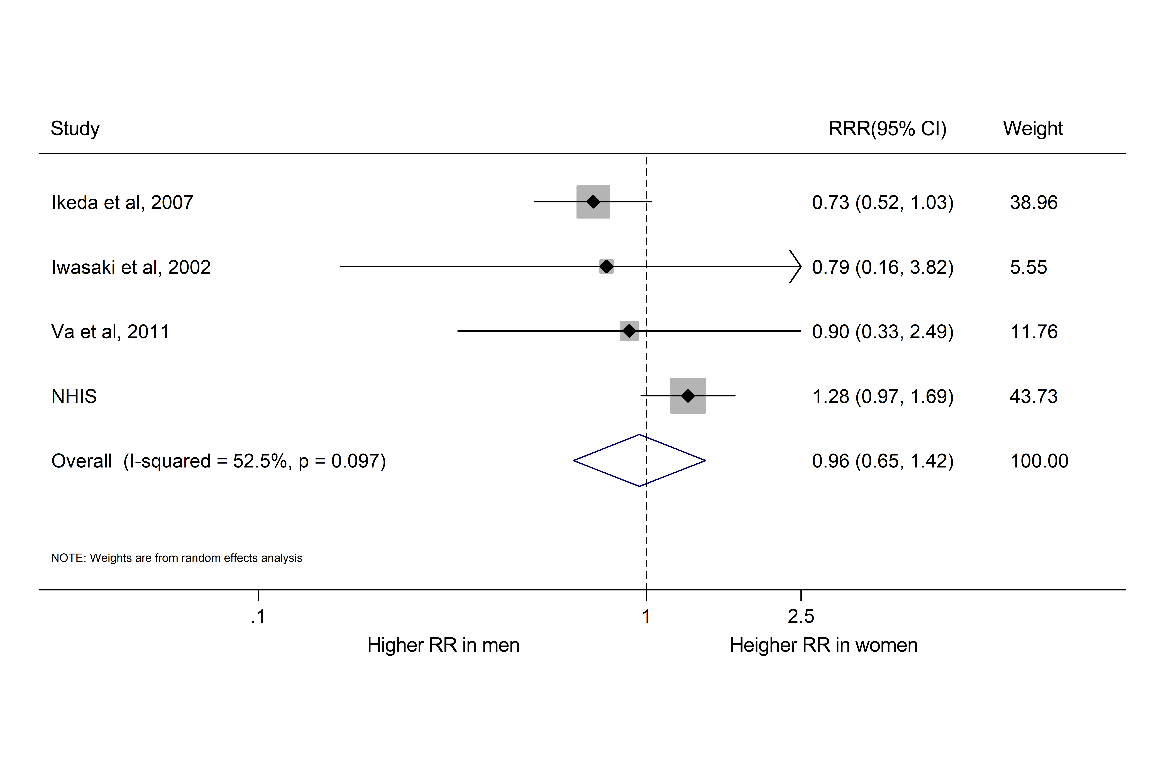
**

**(c) (d)**

**
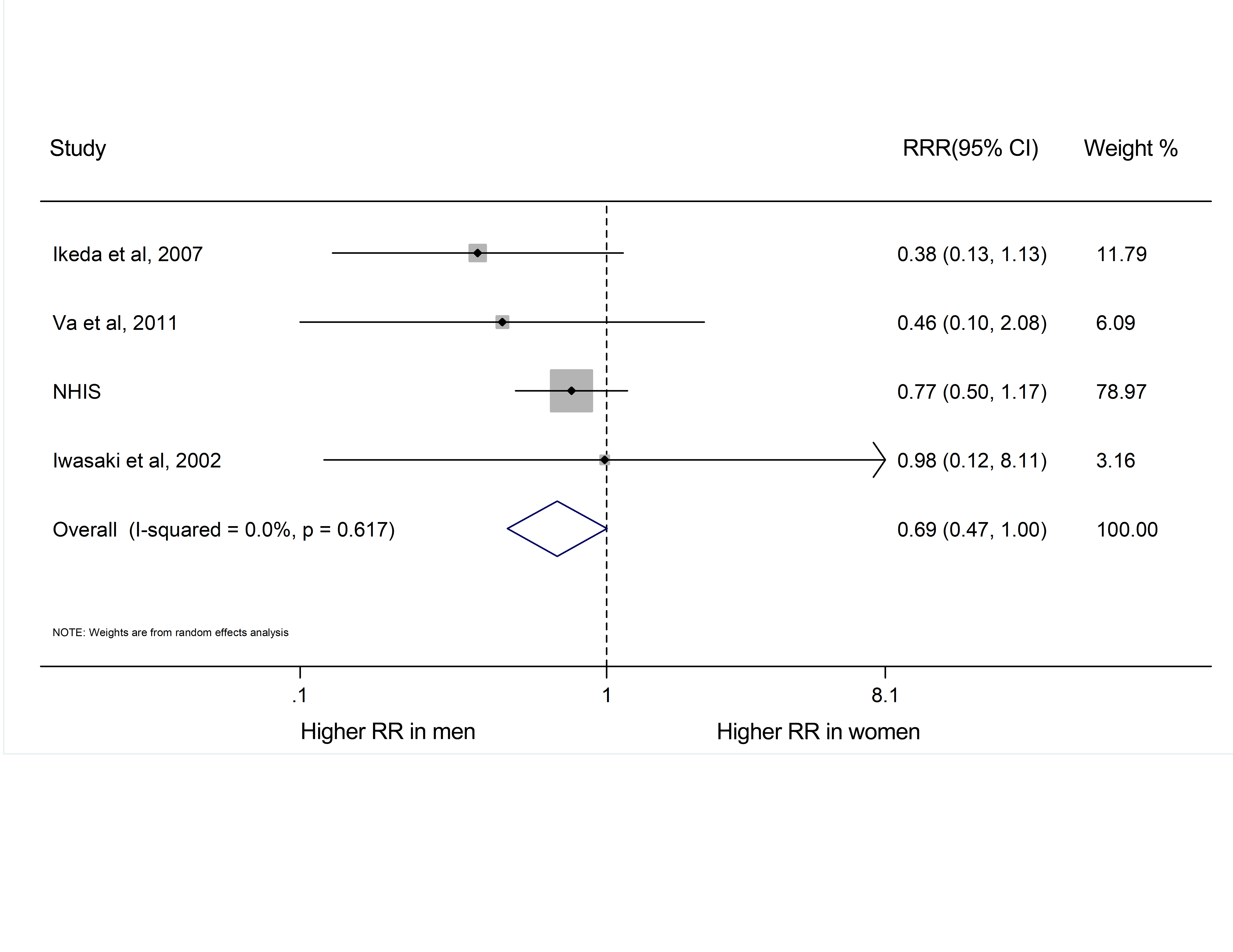

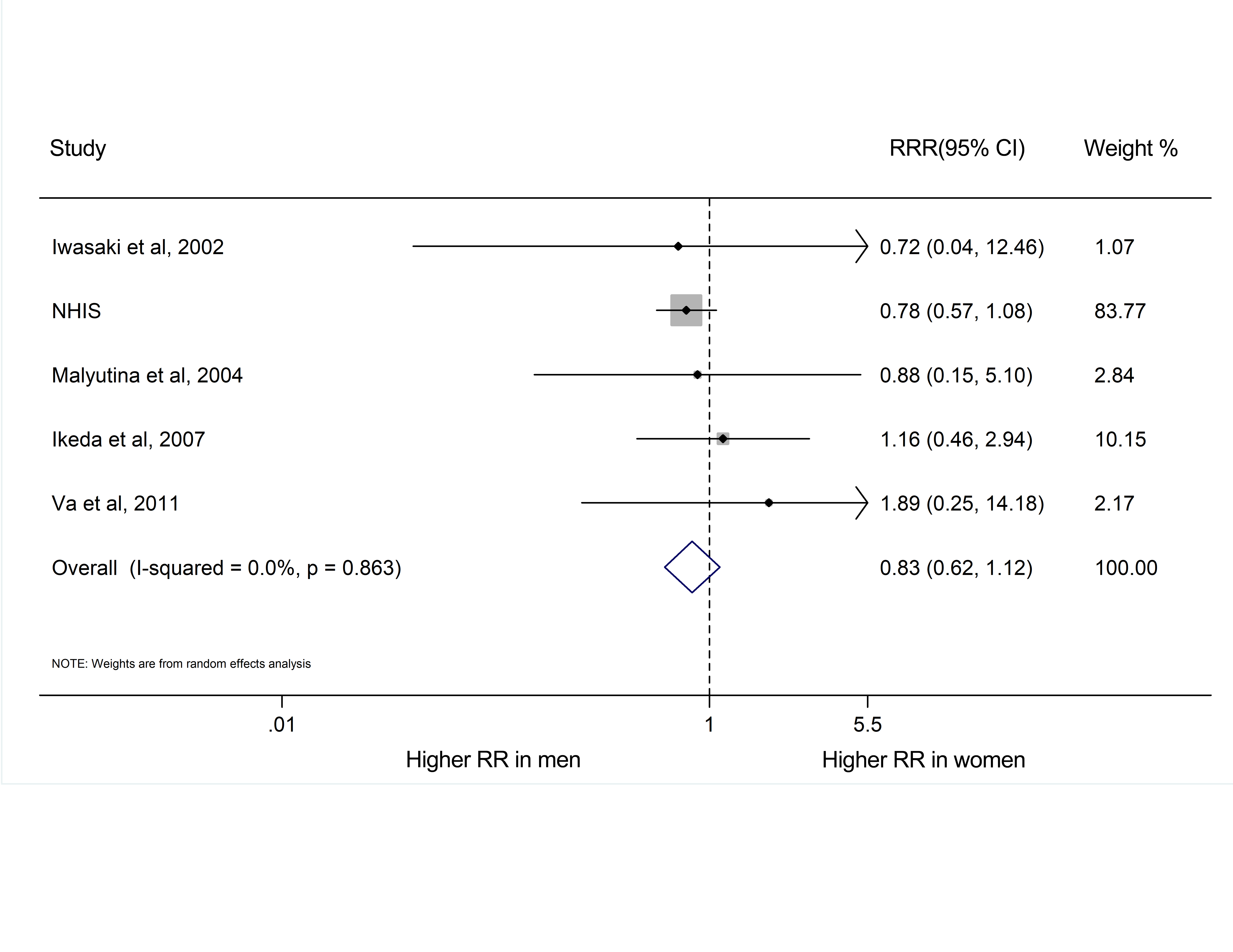
**

**Supplemental Figure 10. Women-to-men ratios of relative risks (RRRs) for stroke mortality comparing non-married, widowed, divorced/separated and never married to married people: (a) Women-to-men RRRs for stroke mortality comparing non-married to married people; (b) Women-to-men RRRs for stroke mortality comparing widowed to married people; (c) Women-to-men RRRs for stroke mortality comparing divorced/separated to married people; (d) Women-to-men RRRs for stroke mortality comparing never married to married people.**


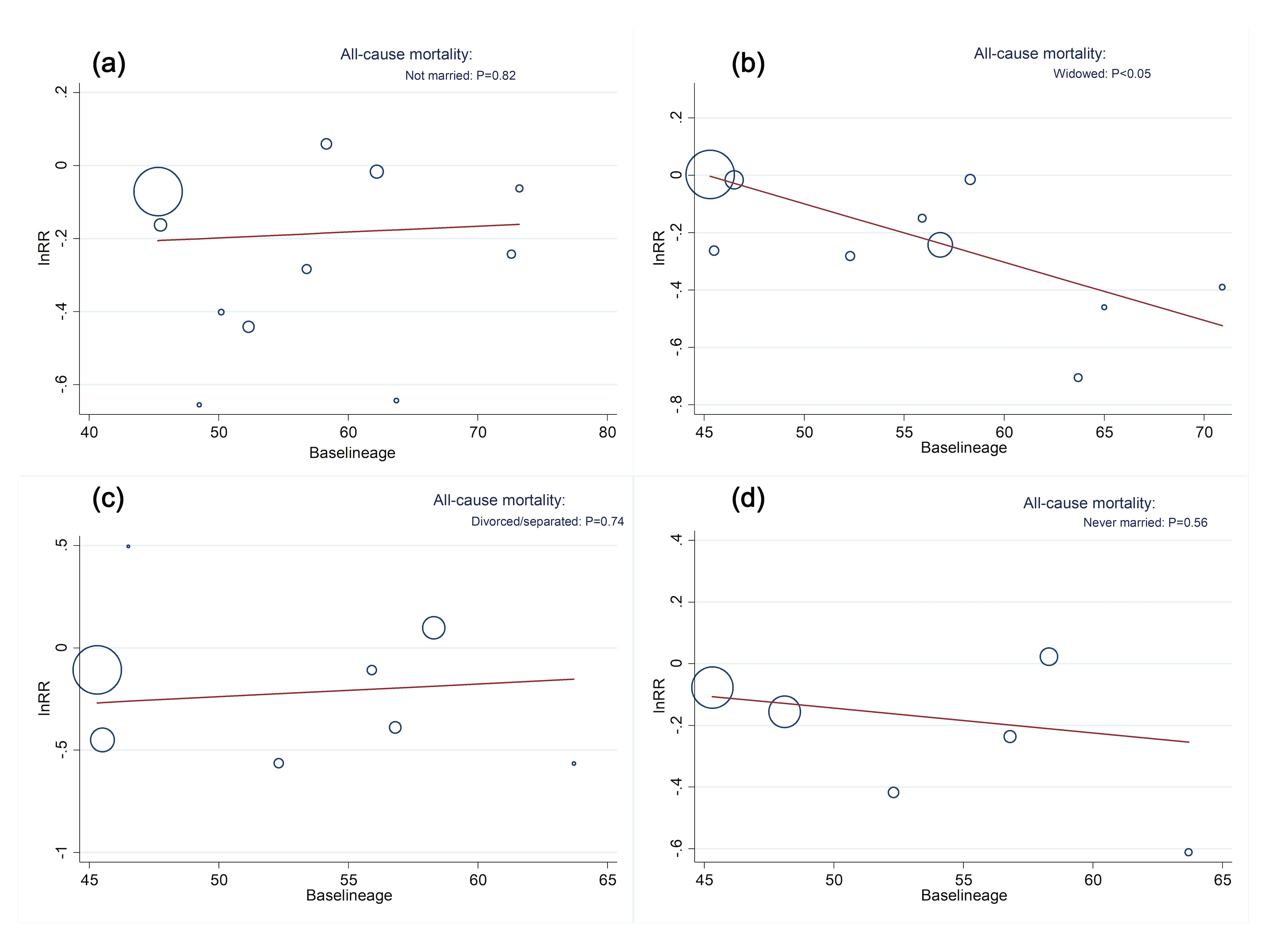


**Supplemental Figure 11. Meta-regression for mean age at baseline for pooled women-to-men ratios of relative risk for risk of all-cause mortality comparing non-married people to married people.**

**
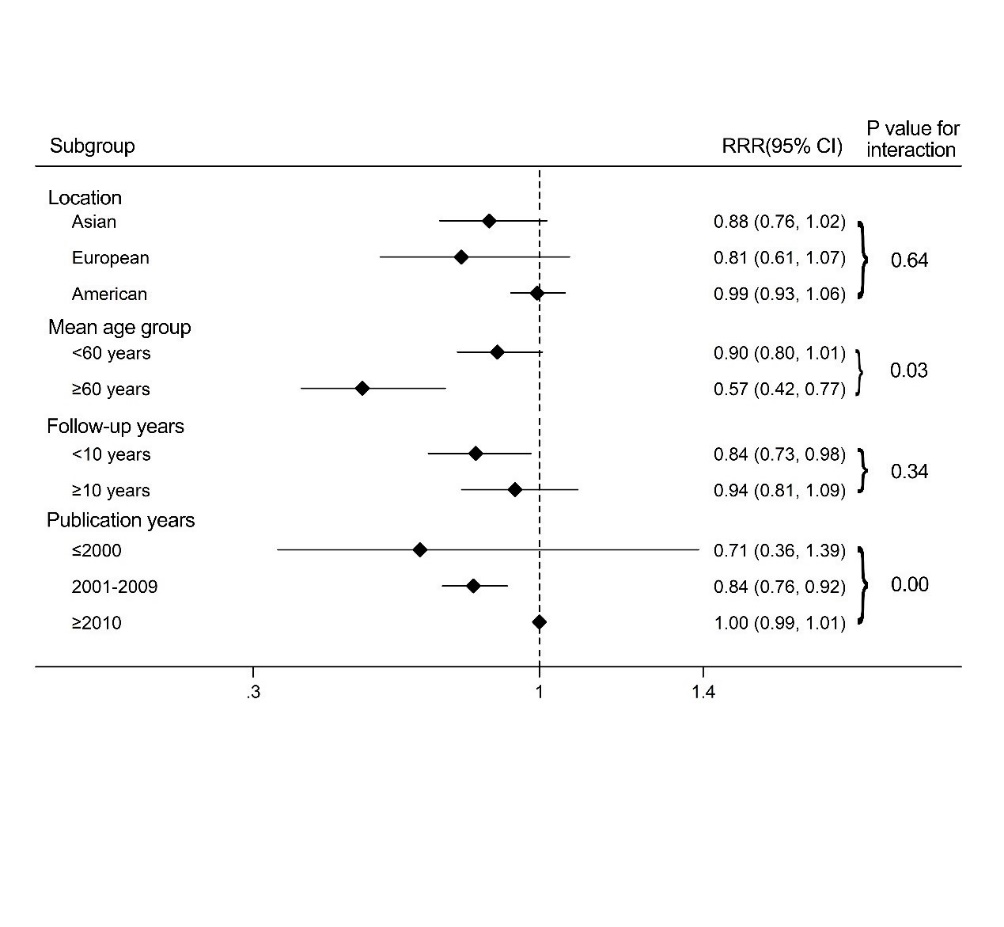
**

**Supplemental Figure 12. Subgroup analyses for all-cause mortality for pooled women-to-men ratios of relative risk (RRRs) for risk of all-cause mortality comparing widowed people to married people.**

**(a) (b) (c)**


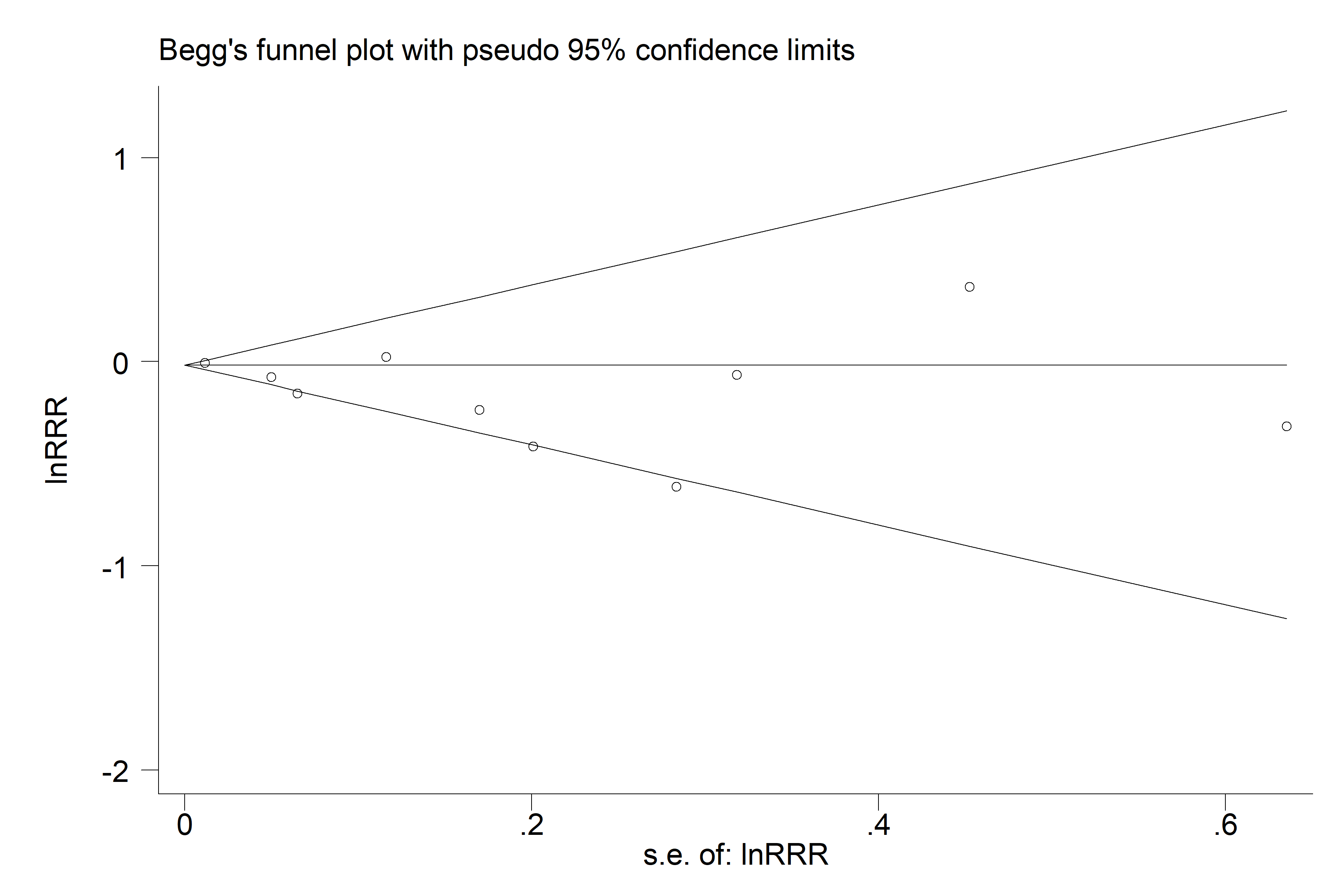

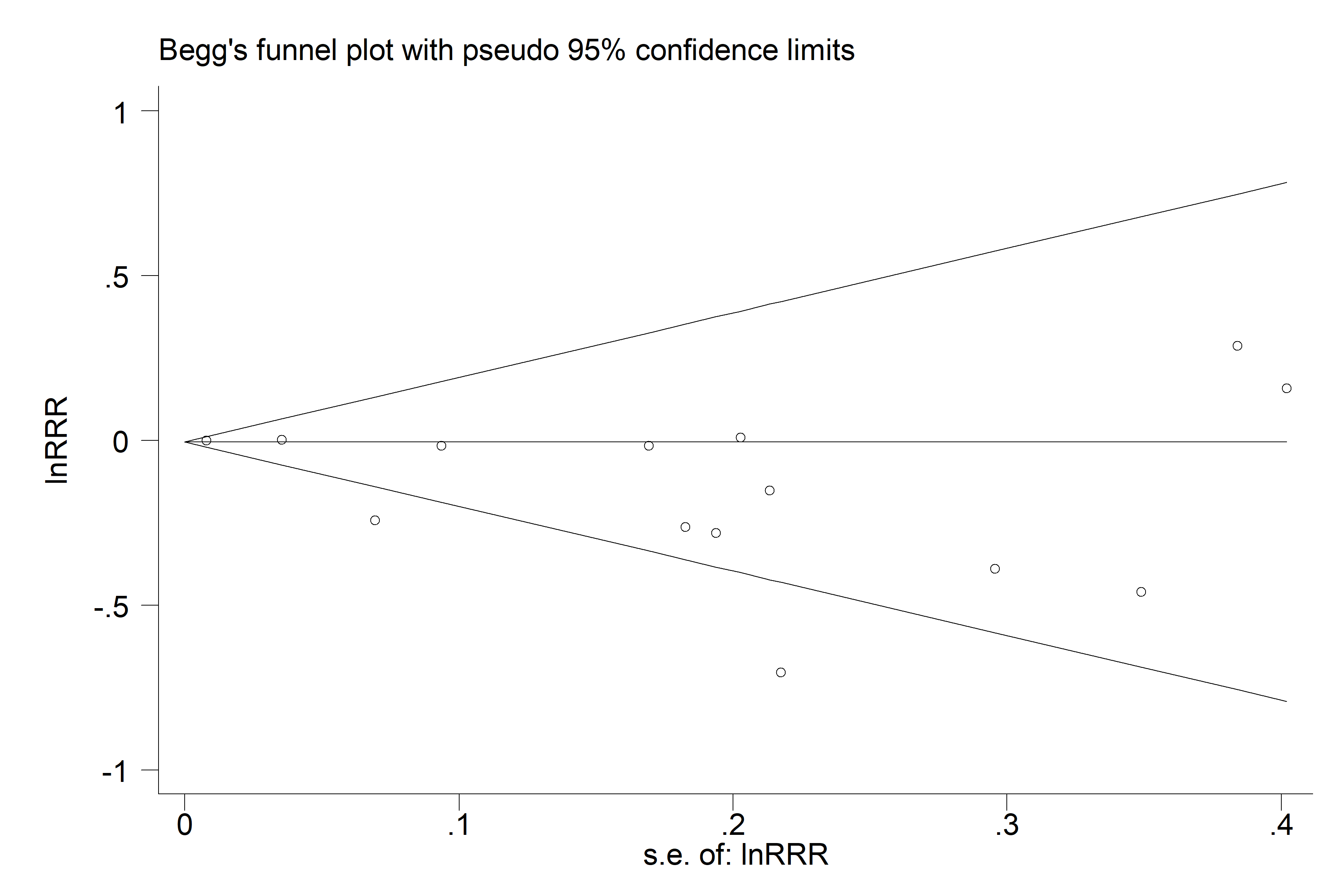

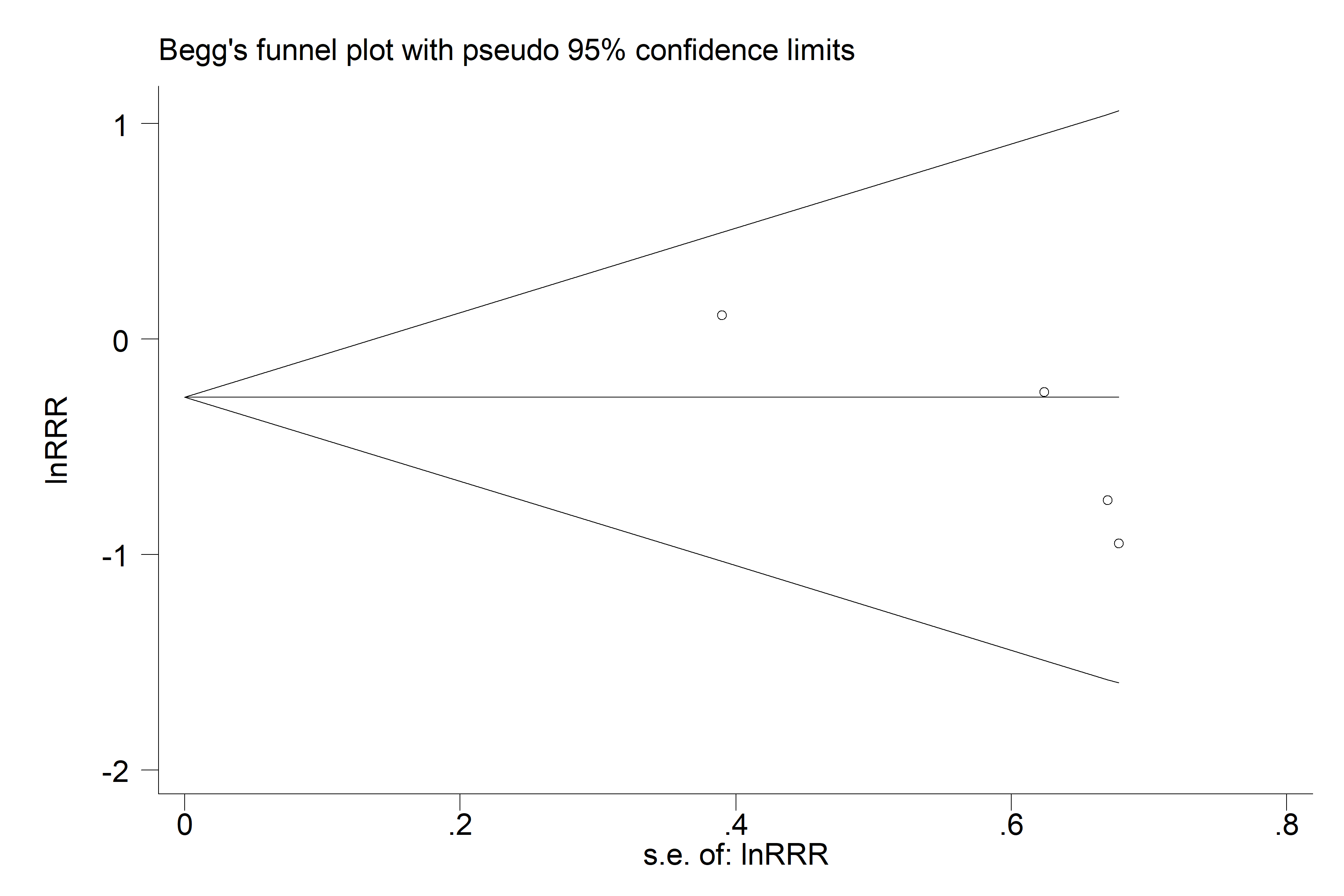


**Supplemental Figure 13.** **Begg’s publication bias plot for the pooled ratio of women-to-men relative risks: (a) Begg’s publication bias plot for CHD mortality in divorced/separated group; (b) Begg’s publication bias plot for all-cause mortality in widowed group; (c) Begg’s publication bias plot for all-cause mortality in never married group; Abbreviations: CHD: coronary heart disease; RR: relative risk; RRR: ratio of RR.**
